# Non-enzymatic heparanase enhances gastric tumor proliferation via TFEB-dependent autophagy

**DOI:** 10.1038/s41389-022-00424-4

**Published:** 2022-08-15

**Authors:** Min Yang, Bo Tang, Sumin Wang, Li Tang, Dalin Wen, Israel Vlodavsky, Shi-Ming Yang

**Affiliations:** 1 Department of Gastroenterology, Xinqiao Hospital, Army Medical University, 400037 Chongqing, China; 2 Wound Trauma Medical Center, State Key Laboratory of Trauma, Burns and Combined Injury, Daping Hospital, Army Medical University, 400042 Chongqing, China; 3 Cancer and Vascular Biology Research Center, the Bruce Rappaport Faculty of Medicine, Technion, Haifa 31096 Israel

**Keywords:** Gastric cancer, Autophagy

## Abstract

Heparanase (HPA) is the predominant enzyme that cleaves heparan sulfate and plays a critical role in a variety of pathophysiological processes. HPA activity has been traditionally correlated with tumor metastasis due to participation in the cleavage and remodeling of the extracellular matrix (ECM). Apart from its well-characterized catalytic properties, HPA was noticed to exert biological functions not rely on its enzymatic activity. This feature is supported by studies showing induction of signaling events, such as Src and AKT, by nonenzymatic HPA mutant. We provide evidence here that active HPA and inactive HPA mutant proteins enhance gastric cancer cell growth, possibly attributed to TFEB-mediated autophagy. Similarly, HPA gene silencing resulted in decreased gastric cancer cell proliferation and autophagy. Besides, TFEB inhibition reduced cell growth and autophagy induced by nonenzymatic HPA. Notably, HPA and TFEB were significantly elevated in gastric carcinomas compared with the adjacent gastric tissue. Moreover, the elevation of HPA gene expression and upregulation of TFEB levels have been associated with advanced clinical stage and poor prognosis of gastric cancer, providing strong clinical support for a connection between TFEB and HPA. Thus, neutralizing the nonenzymatic function of HPA and the related TFEB-driven autophagy may profoundly impact gastric cancer progression.

## Abstract (web summary)

Non-enzymatic HPA reduces mTOR expression and inhibits TFEB phosphorylation, resulting in dissociation of mTOR binding to 14-3-3 protein. The dephosphorylated TFEB is subsequently translocated into the nucleus and activates the expression of autophagy and lysosomal genes which promote the autophagic process and thereby aggravate the growth of gastric cancer cells.

## Graphical abstract

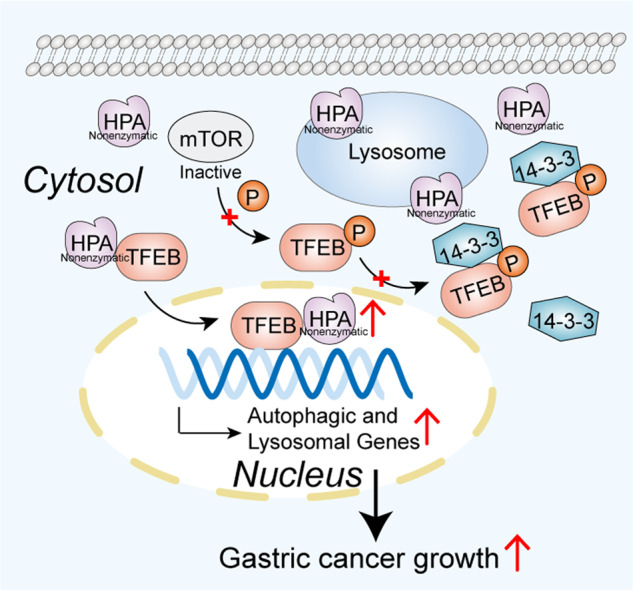

## Introduction

Mammalian heparanase (HPA) is the only endoglycosidase that degrades heparan sulfate, a vital element of the extracellular matrix (ECM) and tumor microenvironment [1, 2]. HPA activity is strongly implicated in the metastatic potential of tumor cells, a result of heparan sulfate degradation and remodeling of ECM barriers [3, 4]. Importantly, Epithelial–mesenchymal transition (EMT) is a key biological process for HPA-derived cells to acquire the ability of migration and invasion. Previous studies have reported that HPA modulates the activity of factors such as FGF-2 and TGF-β-induced EMT [5, 6]. Besides, EMT makers were also found higher in gastric signet ring cell adenocarcinoma with higher HPA expression [7]. Similarly, HPA activity contributes to angiogenesis, inflammation, and autoimmunity [8–10]. Evidence accumulating over the past two decades, indicates that highly expressed HPA exists in the majority of primary tumors, including carcinomas, sarcomas, and hematological malignancies, correlates with larger tumor size, higher microvessel density, and worse prognosis [11, 12]. Apart from its roles as an enzymatic element, heparanase also fulfils enzymatic-independent biological functions, including signal transduction [13, 14] and gene transcription [15, 16]. Activation of serine/threonine kinase (AKT) is regarded as a prominent nonenzymatic signaling event exerted by HPA [17], and its inhibition attenuates cell growth [18]. HPA can also promote cell proliferation by enhancing the phosphorylation of epidermal growth factor receptor, independent of its enzymatic activity [19, 20].

Autophagy is an intracellular catabolic process that degrades cytoplasmic macromolecules and subcellular organelles, essential for cell survival and homeostasis [21]. Autophagy is a dynamic recycling system, consisting of the formation of autophagosomes, fusion of autophagosome–lysosome, and the degradation of autolysosomes [22, 23]. Given the important role of autophagy in cell metabolism, autophagy is closely related to various diseases [24, 25], including cancer [26]. Importantly, autophagosome formation and autolysosome biogenesis are controlled by the transcription factor EB (TFEB) and its family members [27, 28] shown to play a role in tumorigenesis [29–31].

Here we show that HPA enhances gastric cancer progression independently of its enzymatic activity. Moreover, nonenzymatic HPA triggers TFEB-driven autophagy and the associated gastric cancer (GC) cell proliferation. Moreover, upregulation of TFEB was highly correlated with higher expression of HPA and shorter survival of GC patients, thus establishing a feedback loop that drives GC tumorigenicity.

## Materials and methods

### Cells lines and cell culture conditions

The human normal gastric cell line (GES-1) and six gastric cancer epithelial cell lines (Supplementary Table S1) were obtained from the Chinese Academy of Sciences. Cells validation using short tandem repeat markers (STR) were performed by Meixuan Biological Science and Technology Ltd. (Shanghai) or by Biowing Applied Biotechnology Co. Ltd. (Shanghai). All cells were grown according to the instruction. Hyclone cell culture reagents (Hyclone, USA) supplemented with 10% FCS (BioInd, 040011AC5) were used in the cell culture experiments. The cell incubator was humidified with 5% CO_2_, and the temperature is 37 °C. Avoid repeated freezing and thawing of cells.

### Reagents

The PG545 was provided by Dr. Edward Hammond (Zucero Ltd., Brisbane, Australia) [32]. PG545 is a potent HPA inhibitor that inhibits the catalytic activity of HPA by binding to a site adjacent to the hydrophobic region of the HPA active site. And PG545 is also a potent angiogenesis inhibitor by sequestering angiogenic growth factors in the ECM, thereby limiting subsequent binding to receptors [33]. 3MA and CQ were both purchased from Sigma-Aldrich, and Lyso-Tracker Red was obtained from beyotime.

### Plasmids and RNA interference

Plasmids (HPA1, E225/343A mutant, and HPA knockdown) were obtained from Gene (Shanghai, China). GFP-LC3 plasmid and Premo™ Autophagy Tandem Sensor RFP-GFP-LC3B Kit were purchased from Cell Biolabs and Life Technologies, respectively. siRNA for TFEB was purchased from Ribobio (Guangzhou, China).

### Cell viability assay

A CCK8 kit (Dojindo, CK04) was used for cell viability analysis, according to the manufacturer’s guidelines. Briefly, 5 × 10^3^ cells per well were inoculated into 96-well plates. Following plasmid or siRNA transfection and starvation, 10 μl CCK8 reagent was mixed into each well for 1 h incubation at 37 °C. The OD at 450 nm was then determined (VarioskanFlash, Thermo Fisher Scientific, Germany).

### Detection of GFP-LC3 or GFP-RFP-LC3 puncta

For autophagy analysis, GFP-LC3 (Cell Biolabs, CBA-401) or GFP-RFP-LC3 (HANBIO, HB-LP2100001) labels in cells were quantified in each cell. The cells were transfected with either control vehicle, HPA, or HPA mutant plasmid for 24 h and starvation for 4 h. Subsequently, cells were imaged and quantified using Leica confocal scanning microscope (Leica, Germany) and microsystems software (LAS AF, TCS MP5).

### Homogeneous time-resolved fluorescence (HTRF) assay for heparanase enzyme activity

Briefly, 5 μl of supernatants were collected into microtubes for the HTRF assay. After pre-incubation, HPA enzymatic responses were primed by adding 6.0 μl of Bio-HS-Eu(K) (Cisbio, 61BHSKAA) for 30 min at 37 °C, according to the manufacturer’s instruction. In all, 12 μl of SA-XLent solution (Cisbio, 610SAXLF) were subsequently pipetted into tubes to stop the enzyme reaction. After incubation for 30 min at room temperature (RT), 20 μl of the mix were added to a 96-well plate, which was further read on a compatible HTRF reader (Varioskan_LUX, Thermo Fisher Scientific, Germany). HPA enzymatic activity was detected by analysis of the OD ratio of 665 nm/620 nm.

### Dual-luciferase reporter assay

A luciferase reporter assay system was used to analyzed the luciferase activities. Briefly, TFEB 3’-UTR-luciferase reporter vectors were created by Sangon Biotech (Shanghai). cells were co-transfected with the firefly luciferase expression vector (TFEB reporter construct) and a *Renilla* luciferase plasmid (a pRL-TK control vector; Promega) using Lipofectamine transfection reagent (Thermo Fisher Scientific, L3000015). After transfection for 24 h, either control, HPA, or HPA knockdown or HPA mutant plasmids was further transfected, and starvation for 4 h. and the dual-luciferase reporter assay were conducted following the user manual (Promega, E1910). The relative luciferase activity was calculated by analyzing the OD ratio of firefly fluorescence/Renilla fluorescence.

### Confocal laser-scanning microscopy

EdU staining was used to detect cell proliferation. Cells cultured on coverslips were fixed in paraformaldehyde (Beyotime, P0099) and permeabilized with 0.5% Triton X-100 (Beyotime, P0096). Subsequently, Edu staining kit (GeneCopoeia, A005) was carried out according to the manufacturer’s protocol. Cells were then visualized under a Leica microscope, as described above.

To analyze the subcellular location of TFEB and TFE3 transcription factors, the fixed and permeabilized cells were then incubated with monoclonal anti-TFEB or anti-TFE3 antibodies with or without anti-HPA antibody. The cells were then incubated with either anti-mouse antibody (Beyotime, A0428) or anti-rabbit antibody (Beyotime, A0468, or A0516) for 1 h. Subsequently, DNA staining (Beyotime, C1005) for 2 min and Leica confocal laser-scanning microscopy were performed as described above.

To assess autophagy, cells were transfected with a GFP-LC3 or GFP-RFP-LC3 plasmid, as mentioned above. After subsequent transfection with HPA wild-type or mutant plasmid, cells were subjected to confocal scanning microscopy.

To explore the effect on lysosome formation, Lyso-Tracker Red probe (Beyotime, C1046) staining was performed for 30 min at RT. Cells were then imaged under a Leica microscope, as described above.

### Colony-formation assay

The MGC803 cells transfected with NC or wild-type HPA or HPA mutant plasmid for 48 h were plated in 6-well plates at a density of 300 cells per well. And the medium was changed every 3 days. After incubation for 14 days, the colonies were stained with crystal violet (C0121, Beyotime) for 10 min and rinsed with distilled water. Cells were then visualized under a Leica microscope, as described above.

### PCR analysis

Total RNA was harvested and extracted from GC cells with RNAiso Plus (TaKaRa, 9108) and reversed to cDNA. SYBR Green RT-PCR (TaKaRa, RR047A) was then performed using the Applied Biosystems 7300 Real-Time PCR System (Life Technologies, USA). Each cDNA (50 ng) was amplified using 10 μM of specific primers according to the instruction. PCR primer sequences used are listed in Supplementary Table S2. Relative gene expression of candidate genes was calculated by using β-actin as a control reference gene.

### Immunoprecipitation and western blotting

Immunoprecipitation was performed using Universal Magnetic Co-IP Kit ((Activemotif, 54002), according to the manufacturer’s instructions. Briefly, Cells were lysed with complete whole-cell lysis buffer, and followed by immunoprecipitation (IP) with anti-TFEB monoclonal antibodies and protein-G Sepharose beads. Cell extracts were then subjected to 10% sodium dodecyl sulfate-polyacrylamide gels and western blot analysis using anti-TFEB or phospho-TFEB (Ser211) or HPA or 14-3-3 monoclonal antibodies, followed by secondary peroxidase-conjugated antibodies.

For immunoblotting, cells were lysed with RIPA lysis buffer (Beyotime, P0013) on ice and centrifuged at 4 °C. Next, a BCA Protein Assay Kit (Beyotime, P0012) was used to determine the protein concentration, according to the manual. Subsequently, western blotting assay was conducted as described previously [34]. The primary antibodies and secondary antibodies for western blot was listed in Supplementary Table S3.

### Nuclear and cytoplasmic fractionation

The isolation of the nucleus and cytoplasm was performed with a nuclear and cytoplasmic fractionation kit (Thermo Scientific, 78833), according to the manufacturer’s protocol. The cytoplasmic and nuclear fractions were subjected to SDS-PAGE, and protein levels of TFEB were analyzed using western blotting. Lamin B1 and GAPDH were used as nuclear and cytoplasmic markers, respectively.

### Immunohistochemistry and expression profiling in the TCGA dataset

HPA and TFEB expression were detected by GC tissue microarrays containing fifteen paired GC and peritumoral tissue samples (Outdo Biotech, HStmA030PG05). Briefly, 4-μm sections of paraffin-embedded samples were stained with primary anti-HPA or anti-TFEB antibodies, using the Elivision^TM^ plus Plyer HR IHC kit (Maxim, KIT-9901). Images of representative fields were obtained using Olympus fluorescence microscope.

UALCAN (http://ualcan.path.uab.edu/index.html) is an online resource designed for analyzing cancer OMICS data (TCGA, MET500, and CPTAC). We utilized this tool to evaluate the link between HPA expression and the progression of gastric cancer. Meanwhile, Kaplan–Meier plotter (http://kmplot.com/analysis/), an online tool that is capable of verifying the effect of multiple genes on patient survival, was used to assess the relationship between TFEB expression and the outcome of gastric cancer patients.

### Statistical analysis

The UALCAN and Kaplan–Meier plotter online tools were used to generate figure using their separated statistical methods, such as HR and *P* values, and *P* values derived from a log-rank test. All data are shown as means ± SEM. Statistical analysis was performed using GraphPad Prism 8.2.1 software. Student’s *t* test/one-way ANOVA analysis was used to compare the differences. A value of *P* ≤ 0.05 was considered statistically significant.

## Results

### Nonenzymatic heparanase (HPA) enhances gastric cancer cell proliferation

To understand the expression of HPA in GC cell lines, we first compared HPA expression levels among six GC epithelial cell lines (AGS, MKN45, SGC7901, MKN74, MGC803, BGC823) and a normal gastric cell line (GES-1). RT-PCR analyses revealed that HPA was expressed to various degrees in the aforementioned cell lines (Supplementary Fig. 1a), which was further confirmed at the protein level by western blot analysis (Supplementary Fig. 1b). Compared with the GES-1 cell line, MKN45 cells revealed the highest expression of HPA while MGC803 cells showed the lowest expression. Similar results were obtained when cell lysates were subjected to homogeneous time-resolved fluorescence (HTRF) assay of HPA enzymatic activity (Supplementary Fig. 1c), reflecting an obvious heterogeneity among the gastric cancer cell lines [35]. MKN45 and MGC803 cells showed the highest and lowest HPA expression levels, respectively, Thus, we selected these two cell lines for further molecular and cell biology studies.

**Fig. 1:**
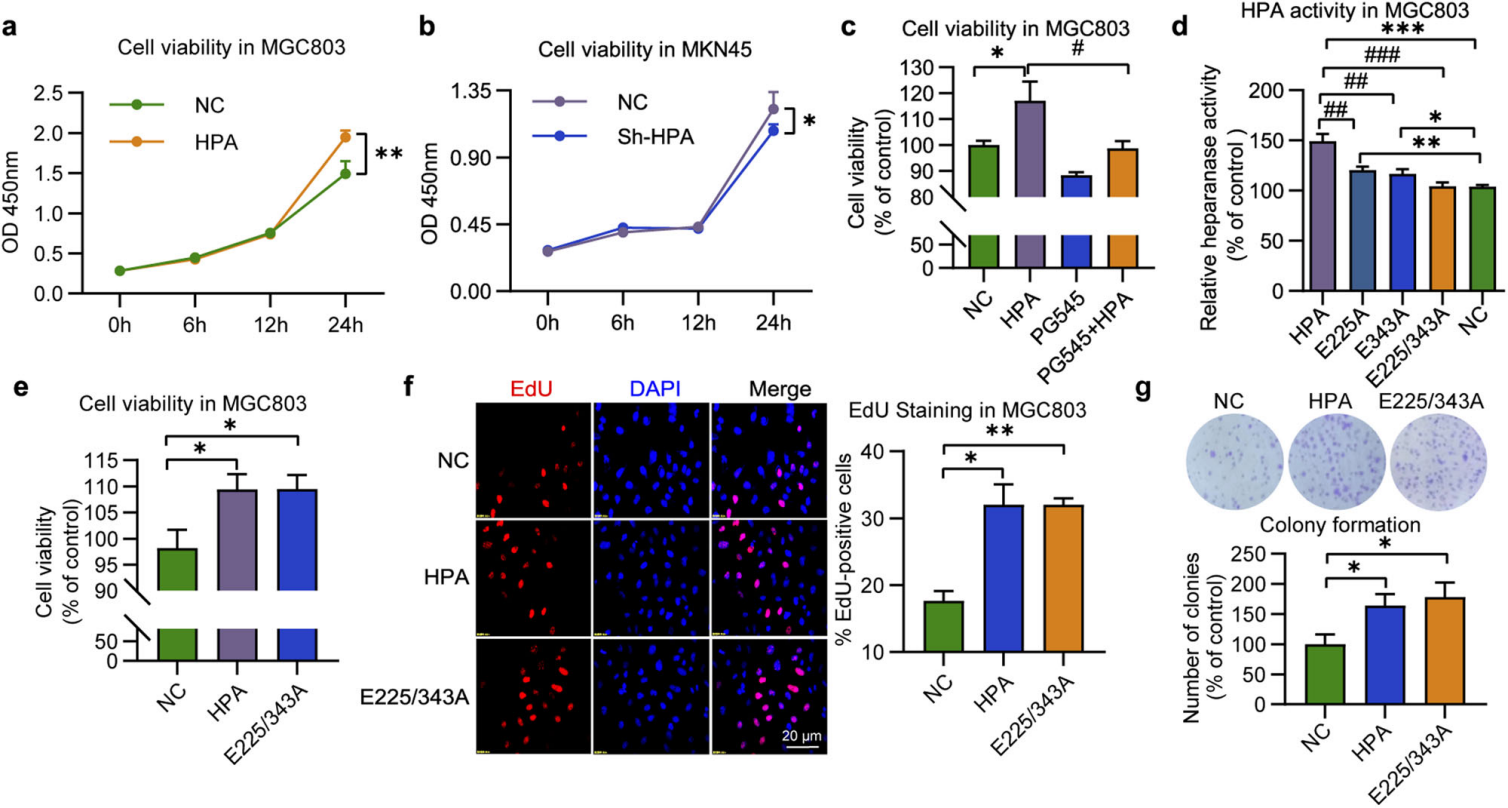
Nonenzymatic HPA induces cell proliferation in gastric cancer cells. Proliferation of HPA-overexpressing MGC803 cells (**a**) and HPA-knockdown MKN45 cells (**b**). **c** Effect of PG545 on the proliferation of HPA-overexpressing MGC803 cells. **d** HPA enzymatic activity in MGC803 cells after transfection with active or inactive HPA. Cell viability (**e**) and EdU staining (**f**) and colony formation (**g**) of MGC803 cells overexpressing active or inactive HPA. *P < 0.05, **P < 0.01, ***P < 0.001 versus the NC group, ^#^*P* < 0.05, ^##^*P* < 0.01, ^###^*P* < 0.001 versus the HPA group.

To evaluate the association between HPA expression levels and cell proliferation, we subjected MGC803 cells to HPA overexpression and MKN45 cells to HPA gene knockdown, and the transfection results were verified at the mRNA and protein levels (Supplementary Fig. 1d, e). The transfected cells were then examined for cell viability. While HPA overexpression increased cell viability (Fig. 1a), HPA knockdown decreased it (Fig. 1b). Notably, the increased cell viability obtained in response to HPA overexpression was prevented by pretreatment with the heparanase inhibitor PG545 (Fig. 1c), indicating that HPA promotes GC cell proliferation. Apart from the well-studied enzymatic feature of the enzyme, HPA can also exert pro-tumorigenic functions independent of its enzymatic activity [36, 37]. To elucidate whether the enhanced GC cell proliferation requires the enzymatic activity of HPA, MCG803 cells were transfected with HPA mutated at the active site amino acid 225 or 343 alone (E225A or E343A) or together (E225/343A double mutant). qPCR and western blotting confirmed that the mutated forms of HPA were expressed both at mRNA and protein levels (Supplementary Fig. 1f). As expected, the activity assay revealed that the transfected cells were devoid of HPA enzymatic activity as compared with mock-transfected cells (Fig. 1d). Subsequently, we examined the viability of MGC803 cells transfected with the double mutant and found that nonenzymatic HPA improved their viability as well (Fig. 1e). Moreover, Edu staining further confirmed that nonenzymatic HPA promotes the proliferation of MGC803 cells (Fig. 1f). Consistently, overexpression of both naïve and mutant HPA caused a clear increased in the clonogenic potential of MGC803 cells (Fig. 1g). Taken together, these results demonstrate that HPA enhances GC cell viability and proliferation independent of its enzymatic activity. In addition, since EMT plays an important role in HPA-mediated tumor development, we further assessed whether EMT markers were altered in our model. The results suggested the EMT maker included Snail and Slug, but not Vimentin, were significantly increased in cells overexpressing wild-type or mutant HPA (Supplementary Fig. 2a), suggesting that EMT may also play a key role in nonenzymatic HPA-mediated gastric cancer development.

### Nonenzymatic HPA enhances gastric tumor cell growth by augmenting autophagy

**Fig. 2:**
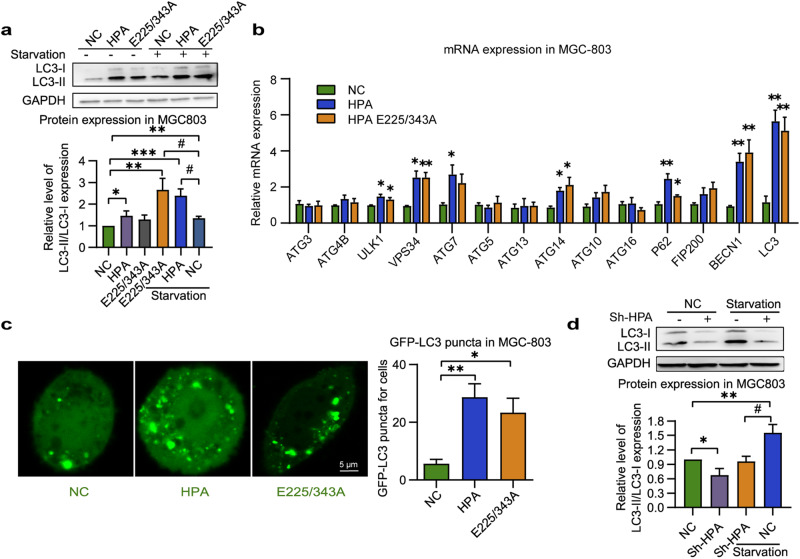
Autophagosome formation in MGC803 cells overexpressing wild-type and nonenzymatic HPA. **a** Western blot analysis of LC3-II in MGC803 cells overexpressing native or mutant HPA, in the absence or presence of starvation. Glyceraldehyde-3-phosphate dehydrogenase (GAPDH) was used as a loading control. **b** Autophagosome formation-related gene expression in MGC803 cells overexpressing native or mutant HPA with deprivation of nutrients for 4 h. **c** Formation of GFP-LC3 puncta analyzed and quantified in MGC803 cells overexpressing native or mutant HPA and subjected to starvation. **d** Representative immunoblot of LC3-II in MKN45 cells after HPA gene knockdown and starvation. GAPDH was used as a loading control. *P < 0.05, **P < 0.01, ***P < 0.001 versus the NC group, ^#^*P* < 0.05 versus the NC + starvation group.

Autophagy is typically induced by stress and nutrient starvation, which contribute to cancer cell survival by maintaining energy homeostasis to meet the elevated metabolic demand [38]. To verify the effect of nonenzymatic HPA on autophagy, cells were deprived of nutrients (starvation) for 4 h to stimulate autophagy after transfection. Autophagosome formation is an important sign of autophagy and can be measured by the conversion from endogenous LC3-I to LC3-II [39]. Notably, transfection of either native or nonenzymatic HPA upregulated the abundance of LC3-II protein in MGC803 cells exposed to nutrient starvation (Fig. 2a). Given that multiple autophagy genes are involved in the initiation and regulation of autophagy [40, 41], we next detected gene expression involved in autophagosome formation. It was found that overexpression of native or mutant inactive HPA enhanced the abundance of autophagosome formation-related genes (including ULK1, VPS34, ATG7, ATG14, P62, BECN1, and LC3) (Fig. 2b) in the condition of starvation. Moreover, image analysis was performed to measure the level of GFP-LC3-positive autophagosomes in cells overexpressing wild-type or mutant HPA after starvation. The results indicate that the cytosolic number of GFP-LC3 puncta per cell improved about fivefold and threefold after HPA and mutant HPA transfection, respectively, compared with mock-transfected cells (Fig. 2c). In addition, deprivation of nutrients increased the LC3-II protein level, as opposed to HPA knockdown in MKN45 cells (Fig. 2d). Similar results were obtained with SGC7901 cells subjected to HPA knockdown (Supplementary Fig. 2b). These data suggest that HPA overexpression promotes autophagosome formation independent of its enzymatic activity.

### Autophagy inhibition reduces GC cell proliferation induced by nonenzymatic HPA

**Fig. 3:**
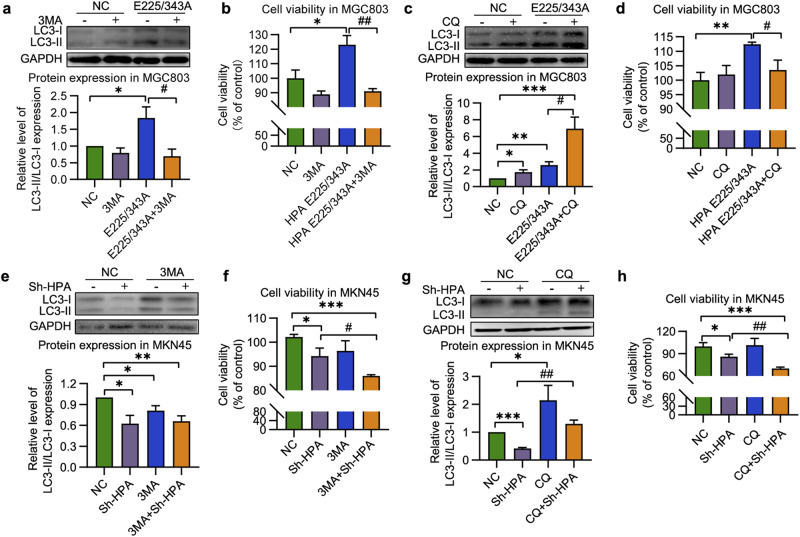
LC3 protein abundance and cell viability induced by nonenzymatic HPA and reversion by autophagy inhibitor. **a** Western blot analysis of LC3-II and (**b**) Viability of MGC803 cells after transfection with nonenzymatic HPA with or without 3MA. **c** Representative image and quantification of LC3-II protein level, and (**d**) proliferation of MGC803 cells overexpressing HPA mutant in the absence or presence of CQ. **e** Level of LC3-II and (**f**) viability of HPA-knockdown MKN45 cells with or without 3MA treatment. LC3-II protein abundance (**g**) and viability (**h**) of MKN45 cells after HPA-knockdown in the presence and absence of CQ. *P < 0.05, **P < 0.01, ***P < 0.001 versus the NC group, ^#^*P* < 0.05, ^##^*P* < 0.01 versus the HPA E225/343A double mutant group or Sh-HPA group.

To further elucidate the relationship between nonenzymatic HPA and autophagy in GC, autophagy inhibitors were applied followed by transfection of native or nonenzymatic HPA. We first used 3MA, an inhibitor that inhibits autophagy initiation and subsequent autophagosome formation [42, 43]. We found that LC3-II protein abundance was markedly reduced after 4 h of incubation with 3MA (Fig. 3a and Supplementary Fig. 3a) accompanied by attenuation of cell viability (Fig. 3b and Supplementary Fig. 3b). We then examined the effect of CQ, an inhibitor that disrupts autophagosome–lysosome fusion and causes autophagosome accumulation [44, 45]. Clearly, CQ significantly aggravated the upregulation of LC3-II in response to native or nonenzymatic HPA (Fig. 3c and Supplementary Fig. 3c) but also led to a decrease in cell proliferation (Fig. 3d and Supplementary Fig. 3d). Next, we examined the effect of autophagy inhibitors on MKN45 cells that were first subjected to HPA knockdown. Not surprisingly, 3MA remarkably inhibited both LC3-II protein expression (Fig. 3e) and cell viability (Fig. 3f), whereas CQ restored the levels of LC3 (Fig. 3g) but remarkably inhibited cell proliferation (Fig. 3h). These results further indicate that cell proliferation stimulated by nonenzymatic HPA may be attributed to autophagy that takes place in the GC cells.

### Nonenzymatic HPA induces lysosomal biogenesis

**Fig. 4:**
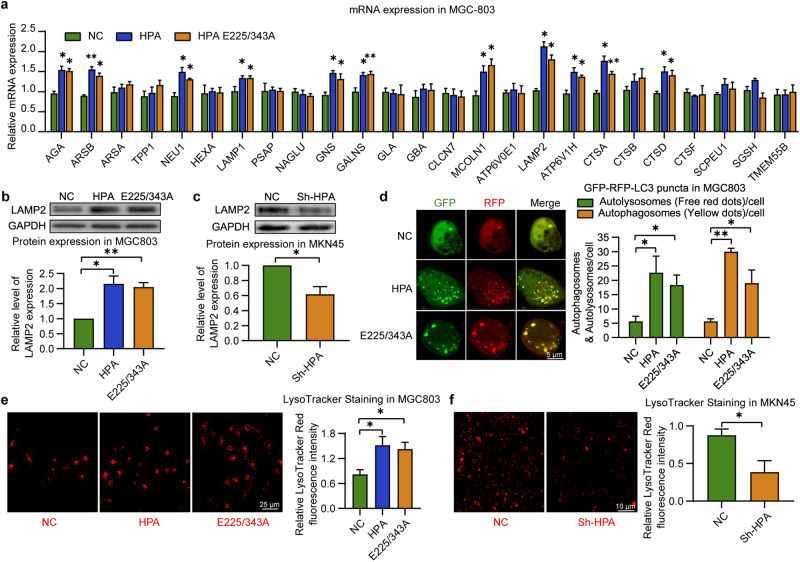
Wild-type and mutant HPA stimulate autolysosome formation in MGC803 cells. **a** lysosome biogenesis-related gene expression in MGC803 cells overexpressing native or mutant HPA and subjected to starvation for 4 h. **b** representative image of LAMP2 protein in MGC803 cells overexpressing native or mutant HPA and subjected to starvation for 4 h. **c** representative immunoblot analysis of LAMP2 in MKN45 cells subjected to HPA gene knockdown and starvation. **d** formation and quantification of GFP-RFP-LC3 puncta in MGC803 cells overexpressing native or mutant HPA in the absence of nutrients. Imaging of lyso-Tracker Red labeled lysosomes in starved MGC803 cells overexpressing native or mutant HPA (**e**), and in MKN45 cells subjected to HPA-knockdown and starvation (**f**). *P < 0.05, **P < 0.01 versus the NC group.

We found that CQ increased the levels of LC3-II and inhibited the proliferation of cells transfected with nonenzymatic HPA. These results suggest that CQ induced autophagy in MGC803 cells, but not inhibited autolysosome fusion. This phenotype prompted us to further understand the role of autophagy in this experimental model. We examined the expression of lysosomal biogenesis genes in MGC803 cells expressing nonenzymatic HPA and subjected to starvation. As expected, nonenzymatic HPA markedly upregulated genes involved in autophagy and lysosomal biogenesis, including those that encode subunits of lysosomal transmembrane proteins (AGA, ARSB, NEU1, LAMP1, GNS, GALNS, MCOLN1, LAMP2), V-ATPase (ATP6V1H) and lysosomal hydrolases (CTSA, CTSD) (Fig. 4a). Moreover, both native and mutant HPA enhanced the expression of LAMP2 in MGC803 (Fig. 4b), and BGC823 (Supplementary Fig. 4) GC cells. As expected, HPA knockdown reduced LAMP2 protein levels in MKN45 cells (Fig. 4c), suggesting that nonenzymatic HPA triggers autophagy and lysosomal biogenesis in MGC803 cells. To assess that nonenzymatic HPA induces autophagy in-depth, MGC803 cells were pretreated with Tandem Sensor RFP-GFP-LC3B. In this assay, autophagosomes show both GFP and RFP (yellow when merged), but only the RFP signal is detected in autolysosomes due to the RFP being relatively stable in acidic conditions [46]. As demonstrated in Fig. 4d, we found that both native and nonenzymatic HPA increased the formation of autophagosomes and autolysosomes. We then used the Lyso-Tracker Red to label the cellular acidic compartment. Lyso-Tracker Red staining revealed a significantly increased fluorescence intensity after transfection of MGC803 cells with native or mutant HPA, compared with mock-transfected cells (Fig. 4e), while downregulation of HPA decreased the fluorescence intensity in MKN45 cells (Fig. 4f). Taken together, these data clearly demonstrate that nonenzymatic HPA can enhance cell autophagy by inducing lysosomal biogenesis and formation of autolysosomes.

### Nonenzymatic HPA-induced nuclear translocation of TFEB

**Fig. 5:**
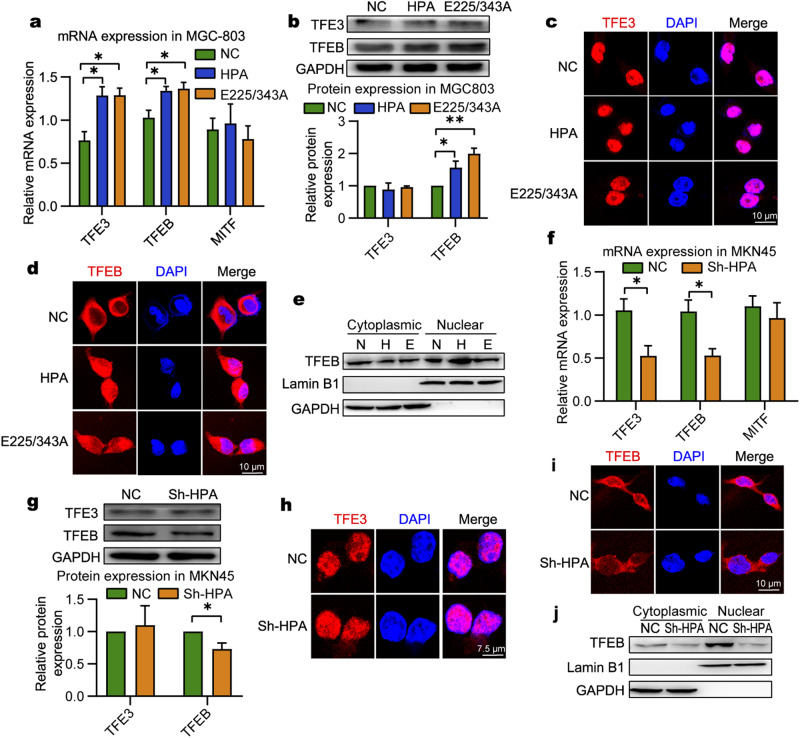
Overexpression of wild-type and nonenzymatic HPA leads to nuclear translocation of TFEB. Relative gene expression of MiT/TFE family members in MGC803 cells overexpressing native or mutant HPA (**a**) or in MKN45 cells subjected to HPA-knockdown (**f**), and starvation. Representative image and quantification of TFE3 and TFEB protein expression in MGC803 cells overexpressing native or mutant HPA (**b**) or in MKN45 cells subjected to HPA-knockdown (**g**), and starvation. Representative image of TFE3 subcellular localization in MGC803 cells overexpressing native or mutant HPA (**c**) or in MKN45 cells subjected to HPA knockdown (**h**), and starvation. Immunofluorescence images of TFEB nuclear translocation in MGC803 cells overexpressing native or mutant HPA (**d**) or in MKN45 cells subjected to HPA knockdown (**i**), and starvation. Cells were subjected to nuclear and cytoplasmic fractionation in MGC803 cells overexpressing native or mutant HPA (**e**) or in MKN45 cells subjected to HPA-knockdown (**j**), and starvation. Protein level of TFEB were analyzed using western blotting. Lamin B1 and GAPDH were used as the nuclear and cytoplasmic markers, respectively. *P < 0.05, **P < 0.01 versus the NC group.

TFEB, together with MITF and TFE3, belongs to the MiT/TFE family of transcription factors, are master genes regulating autophagy and lysosomal biogenesis [47, 48]. We analyzed whether those transcriptional factors are involved in nonenzymatic HPA-induced autophagy in gastric cancer cells. While overexpression of nonenzymatic HPA did not directly affect the level of MiTF, it significantly increased the levels of TFE3 and TFEB (Fig. 5a). Interestingly, a change in protein abundance was observed in TFEB, but not TFE3 (Fig. 5b). Notably, immunofluorescence results showed that both HPA and nonenzymatic HPA had no significant effect on TFE3 translocation (Fig. 5c), but promoted TFEB nuclear translocation (Fig. 5d). Consistent with the results of immunofluorescence, the nuclear and cytoplasmic fractionation assay also showed that both HPA and nonenzymatic HPA decreased the levels of TFEB in the cytoplasm and dramatically increased the levels of TFEB in nucleus (Fig. 5e). Moreover, knockdown of HPA decreased TFEB mRNA and protein levels but had no effect on the expression of TFEB family members (Fig. 5f, g) as well as on TFE3 nuclear translocation (Fig. 5h). However, inhibition of TFEB nuclear entry and decreased level of TFEB in nucleus were observed in response to HPA downregulation in MKN45 cells (Fig. 5i, j). In summary, upregulation of native or nonenzymatic HPA promoted TFEB nuclear translocation in GC cells.

### Nonenzymatic HPA promotes TFEB nuclear translocation in a dephosphorylation-dependent manner

**Fig. 6:**
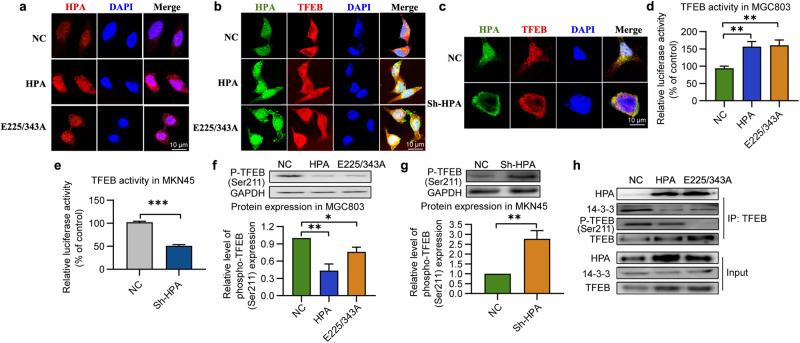
Nonenzymatic HPA activates TFEB in a dephosphorylation-mediated manner. **a** Representative images of HPA subcellular localization in MGC803 cells after transfection with wild-type or mutant HPA. **b** Representative images of HPA and TFEB subcellular colocalization in MGC803 cells overexpressing native or mutant HPA or in MKN45 cells subjected to HPA knockdown (**c**), and starvation. Luciferase activity of TFEB was measured in starved MGC803 cells overexpressing native or mutant HPA (**d**) or in MKN45 cells after HPA knockdown (**e**). Representative immunoblot analysis and quantification analysis of phospho-TFEB(Ser211) in MGC803 cells overexpressing native or mutant HPA (**f**) or in MKN45 cells subjected to HPA knockdown (**g**), and starvation. **h** Cells overexpressing native or mutant HPA were lysed and subjected to immunoprecipitation with anti-TFEB antibody. Immunoprecipitates were analyzed by western blotting with anti-TFEB, or HPA, or phospho-TFEB (Ser211) or 14-3-3 antibodies. *P < 0.05, **P < 0.01, ***P < 0.001 versus the NC group.

In addition to the well-documented perinuclear localization of HPA, several studies reported the existence of HPA in the nucleus and its involvement in the cleavage of nuclear heparan sulfate (HS) and regulation of gene transcription [49, 50]. Here, we report that HPA shuttled into the nucleus after transfection of MGC803 cells with either native or mutant HPA (Fig. 6a). It is worth noting that in both cases, nuclear HPA was co-localized with TFEB (Fig. 6b). In contrast, knockdown of HPA reduced nuclear translocation and colocalization of HPA and TFEB (Fig. 6c). Moreover, luciferase assay showed that TFEB transcriptional activity was increased in response to upregulation of wild-type or mutant HPA (Fig. 6d), and decreased in response to HPA knockdown (Fig. 6e). TFEB nuclear translocation is mainly regulated by energy-sensitive signals, which lead to changes in cell localization by regulating its phosphorylation level [51]. The most notable of these is the mammalian target of rapamycin (mTOR) [52]. It was reported that in head and neck cancer cells, HPA induces autophagy via suppressing mTOR signaling [32]. The mTOR promotes TFEB nuclear translocation primarily by inhibiting its phosphorylation, most prominently at serine 211 (S211) This dephosphorylation promotes the dissociation of TFEB to the chaperone 14-3-3 and entry of the transcription factor into the nucleus [52, 53]. Our results indicate that overexpression of wild-type or mutated HPA significantly inhibits phosphorylation of TFEB at S211 (Fig. 6f), while HPA knockdown enhanced TFEB phosphorylation (Fig. 6g). We further tested whether treatment with native or mutant HPA in MGC803 cells increased the bind of TFEB and HPA and increased the dissociation of 14-3-3 from the TFEB/14-3-3 complex. As expected, the band intensity of HPA protein in TFEB immunoprecipitates was markedly increased after treatment with native or mutant HPA, whereas the bands intensity of p-TFEB at Ser211 and 14-3-3 protein in TFEB immunoprecipitates were significantly reduced (Fig. 6h). These results suggest that nonenzymatic HPA may promote TFEB nuclear entry and transcriptional activity in a dephosphorylation-dependent manner.

### Downregulation of TFEB decreases autophagy and cell proliferation

**Fig. 7:**
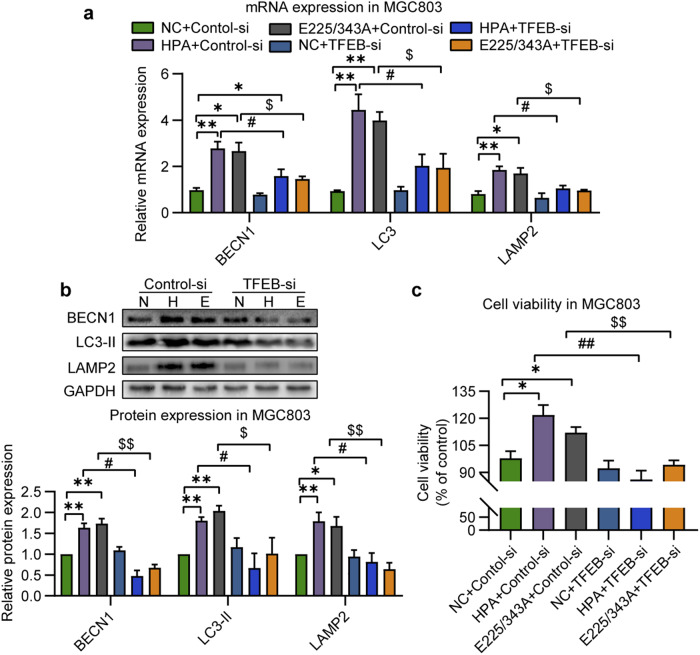
TFEB-mediated autophagy underlies nonenzymatic HPA-mediated cell proliferation. **a** Expression of BECN1, LC3, and LAMP2 in MGC803 overexpressing native or mutant HPA and subjected to TFEB gene silencing. **b** Representative images and quantification of the indicated protein in MGC803 cells overexpressing native or mutant HPA with or without TFEB gene silencing. GAPDH was used as a loading control. **c** Viability of MGC803 cells overexpressing native or mutant HPA and subjected to TFEB gene silencing. *P < 0.05, **P < 0.01 versus the NC group. ^#^*P* < 0.05, ^##^*P* < 0.01 versus the HPA group, ^$^*P* < 0.05, ^$$^*P* < 0.01 versus the E225/343 A group.

TFEB-specific siRNA was applied to further clarify whether TFEB mediates nonenzymatic HPA-induced autophagy in MGC803 cells (Supplementary Fig. 5a, b). As shown in Fig. 7a, b, the expression of HPA/mutant HPA-induced TFEB-responsive genes were effectively suppressed by TFEB gene silencing, including BECN1, LC3, and LAMP2. In addition, overexpression of either wild-type or mutant HPA promoted cell viability, and this protective effect was markedly attenuated by anti-TFEB-siRNA (Fig. 7c). Together, these results demonstrate that nonenzymatic HPA-induced cell proliferation is mediated by activation of the TFEB pathway, and that inhibition of TFEB-dependent autophagy reduces MGC803 cell proliferation.

### Aberrant HPA and TFEB expression in GC tissues

**Fig. 8:**
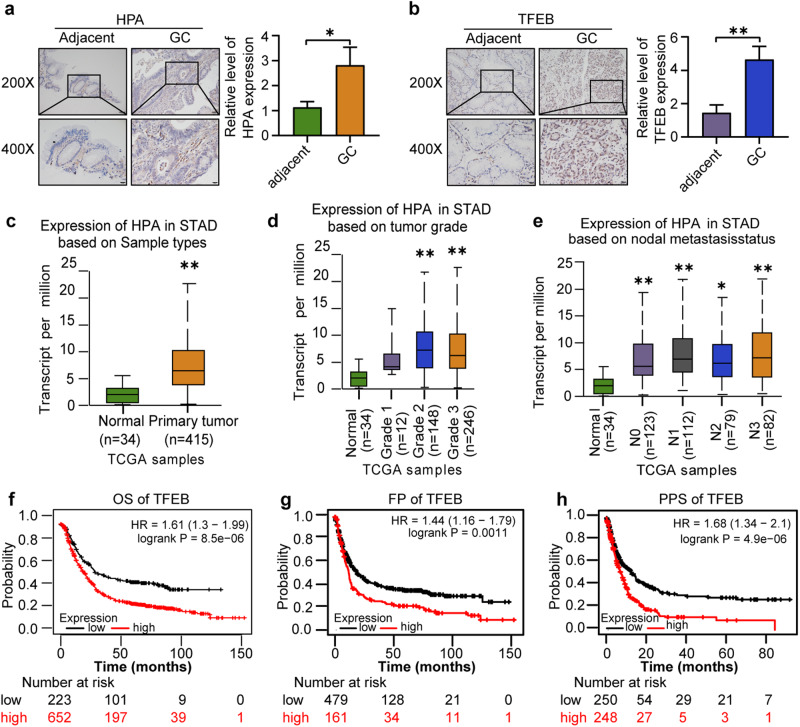
Aberrant expression of HPA and TFEB in gastric cancer. Immunohistochemical analysis of HPA (**a**) and TFEB (**b**) expression in gastric tissues. **c** Relative expression level of HPA in normal gastric tissue and gastric primary tumors from UALCAN database. **d** Relative expression of HPA in gastric cancer based on tumor grade from UALCAN database. **e** expression of HPA in gastric cancer based on lymph nodal metastasis status from UALCAN database. Kaplan–Meier analysis from TCGA database of the correlation between relative expression of TFEB and overall survival (OS) (**f**), first progression (FP) (**g**), or post-progression survival (**h**). *P < 0.05, **P < 0.01 versus the adjacent or normal group. STAD stomach adenocarcinoma.

Aberrant expressions of HPA and TFEB are associated with several human cancers [48, 54–56]. To further analyzed the expression of HPA and TFEB in GC tissues, we used a GC tissue microarray containing 15 pairs of primary GC tissues and adjacent nontumor tissues. Immunohistochemistry was applied to detect HPA and TFEB in human specimens derived from fifteen paired patients with gastric cancer. As shown in Fig. 8a, b, expression of HPA and TFEB was markedly increased in primary GC tissues compared to their adjacent normal-looking tissues. We further analyzed TFEB and HPA in gastric tumors using the TCGA database. Expression of HPA was significantly increased in gastric cancer (Fig. 8c and Supplementary Data 1). Moreover, clinicopathological analysis revealed that the patients with poorly differentiated tumors (Fig. 8d and Supplementary Data 2) and advanced tumor stage (Fig. 8e and Supplementary Data 3) exhibited significantly higher HPA expression. We next applied the Kaplan–Meier analysis and explored the relationship between TFEB expression and the GC patient outcome. The data revealed that TFEB elevation is significantly correlated with poor prognosis of in patients wih GC (OS HR = 1.61, log-rank *P* = 8.5e-06; FP HP = 1.44, log-rank *P* = 0.0011; PPS HP = 1.68, log-rank *P* = 4.9e-06) (Fig. 8f–h). Taken together, these results indicate that increased expression of HPA and TFEB in gastric cancer is correlated with advanced tumor stage and poor prognosis.

## Discussion

Mammalian cells express a single functional endoglycosidase (heparanase, HPA) which degrades heparan sulfate (HS). Cleavage of HS by HPA contributes to the disassembly of the ECM, thereby facilitating cancer metastasis and inflammation. Compelling evidence strongly implies that heparanase serve crucial roles in all aspects of the tumorigenic process, namely tumor initiation, growth, chemotherapy resistance, and metastasis [1–13], pointing to the potential of heparanase as an anti-cancer drug target and incentivizing the development of small molecular inhibitors of heparanase [14, 15]. A critical venue by which heparanase exerts multiple effects on its target tissues and cells is by modulating the bioavailability of HS-bound cytokines, chemokines, and growth factors, initiating the tissue microenvironment. Such architecture allows heparanase-mediated tumor-host crosstalk and promotes several basic cellular processes (i.e., exosome formation, autophagy, immune-inflammatory responses) that together coordinate tissue remodeling [16, 17]. Heparanase functions as an “activator” of HS proteoglycans and therefore is a pivotal player in creating a supportive environment for tumor cell proliferation and dissemination. In addition, it is required for various cellular processes from gene expression regulation to signal transduction and DNA damage signaling [3, 18]. In addition to its enzymatic activity, nonenzymatic functions of HPA have been reported and are well-documented [57, 58]. For example, HPA could augment cell proliferation, mobility, and angiogenesis through activation of β1 integrin, HIF-2α, Flk-1, and/or AKT signaling, independent of its enzymatic activity [17, 18, 59]. In addition, HPA has been documented to impact on the blood clotting system, independent of its catalytic function [60, 61]. Altogether, these and other results indicate that nonenzymatic activities of HPA play essential roles in a variety of pathological processes. Here, we demonstrate that, among other effects, overexpression of enzymatically inactive heparanase promotes GC cell proliferation and autophagy.

Autophagy is a major cellular degradation and recycling process, critical for maintaining cellular homeostasis [62, 63]. Autophagy is a dynamic process that sequesters misfolded and/or potentially dangerous proteins and damaged organelles in double-membranes vesicles called autophagosomes, that are ultimately degraded within lysosomes [64, 65]. The process of autophagy is schematically subdivided into three critical steps: autophagosome formation and maturation, autophagosome–lysosome fusion, and auto-lysosomal acidification [66]. Briefly, the activation of ULK1 and BECN1-VPS34 complexes leads to the formation of autophagosomes [65, 67] that are then fused with lysosomes to produce autolysosomes, followed by intraluminal acidification and subsequent activation of lysosomal hydrolases to mediate autophagic cargo degradation [68, 69]. Given its major role in cellular metabolism, autophagy is connected with numerous disease states [24, 41], yet the role of autophagy in cancer is disputable and context-dependent [24, 26, 48, 70–72]. It has previously been shown that HPA promotes the growth of head and neck carcinoma by enhancing autophagy [32]. In the present study, we found that both active or enzymatically inactive HPA increase autophagosome formation and the expression of related genes in gastric cancer cells. We show that nonenzymatic HPA-induced LC3-II protein expression and cell viability were attenuated by 3MA and CQ, compounds that inhibit autophagosome formation and disrupt autophagosome–lysosome fusion, respectively. We next assessed the biogenesis and formation of lysosomes to further ascertain the role of autophagy in nonenzymatic HPA-induced GC cell proliferation. We observed that both wild-type and mutant HPA increased the expression of autophagy- and lysosomal-related genes as well as the level of the lysosomal membrane protein LAMP2. Fluorescence imaging showed that the formation of autophagosomes, autolysosomes, and lysosomes was increased after transfection with native or nonenzymatic HPA, indicating that nonenzymatic HPA may also trigger cell autophagy by inducing lysosomal biogenesis and formation of autolysosomes in gastric cancer cells.

The MiT/TFE transcription factors are composed of four members: TFEB, TFEC, and TFE3 and MITF. Among them, TFEB and TFE3 are master regulators of lysosomal and autophagosome biogenesis, which bind to a conserved 10-base palindromic sequences, named Coordinated Lysosomal Expression and Regulation (CELEAR) site that promotes the transcription of several lysosomal and autophagy genes [50, 73–75]. A variety of stimuli are related to TFEB and TFE3 activities, among which nutritional deprivation is well-characterized [52, 76, 77]. In the presence of nutrients, TFEB and TFE3 have been shown to reside in the cell cytoplasm, but are rapidly translocated to the nucleus in the absence of nutrients [76, 78]. Subsequently, molecules involved in nutrient sensing and cellular growth have been shown to regulate TFEB and TFE3 activity [43, 79, 80]. The most studied regulatory mechanism of TFEB and TFE3 subcellular localization is involved in modulating the phosphorylation status of multiple serine residues [52]. In particular, mTOR kinase was shown to phosphorylate TFEB and TFE3 and act as a regulatory in TFEB and TFE3 subcellular localization [51, 52]. Several serine residues in TFEB and TFE3 are phosphorylated by mTOR, but S211 in TFEB and S321 in TFE3 are particularly relevant [81–84]. In the presence of adequate nutrition, mTOR phosphorylates these residues to create a cytoplasmic chaperone 14-3-3 binding site. Interaction with 14-3-3 leads to the accumulation of TFEB/TFE3 in the cytoplasm. In contrast, when nutrition is insufficient, inactivation of mTORC1 dephosphorylates S211 and S321 and prevents binding to 14-3-3 leading to rapid TFEB and TFE3 nucleus translocation occurred. The mechanism underlying autophagy induction by HPA is not clear. Shteingauz et al reported that HPA-induced autophagy is mediated by mTOR signaling because HPA overexpression is associated with decreased mTOR activity, whereas HPA inhibition leads to increased mTOR activity and substrate phosphorylation [32]. Here, we report that the mRNA and protein levels of TFEB, but not TFE3, were significantly changed after transfection with either wild-type or nonenzymatic HPA. Moreover, active and mutant HPA promoted nuclear translocation of TFEB but had no significant effect on TFE3 subcellular localization. These data suggest that TFEB- but not TFE3-mediated autophagy is predominant involved in HPA-induced autophagy in GC cells, independent of HPA enzymatic activity. Additionally, upregulation of nonenzymatic HPA decreased phosphorylation of TFEB S211, and abolished the interaction between TFEB and 14-3-3 protein, further indicating that nonenzymatic HPA induces TFEB-mediated autophagy in TFEB dephosphorylation-dependent manner.

Aberrant activation of TFEB is closely related to tumor oncogenesis and development [51, 85, 86]. Cells rely on effective lysosomal function, and increasing evidence indicates that cancer cells may utilize TFEB-dependent transcriptional activation of lysosomal degradation pathways to maintain survival. Overexpression of TFEB was reported to drive pancreatic ductal adenocarcinoma by inducing autophagy [48, 87]. TFEB gene silencing results in a significant decrease in the hyperproliferative phenotype and progression of pancreatic carcinoma to advanced stages [31, 54]. Here, we report that TFEB gene silencing significantly attenuated hyperproliferation induced by nonenzymatic HPA via decreased autophagy and lysosome biogenesis. Moreover, upregulation of HPA in GC is significantly linked to advanced tumor stage, while increased TFEB expression in GC tissues is correlated with poor prognosis. These results further indicate that HPA induces GC progression through TFEB-mediated autophagy, independent of HPA enzymatic activity (Graphical abstract), offering a new strategy for the design of nonenzymatic HPA targeting drugs.

However, our results are mainly derived from gastric cancer cell lines, and it is necessary to repeatedly verify the regulatory relationship between HPA and TFEB in animal in vivo experiments. We will make further improvements in follow-up research.

## Supplementary information

Supplemental figures

Table S1

Table S2

Table S3

Supplemental data 1

Supplemental data 2

Supplemental data 3

author list change

## References

[1] Freeman C, Browne AM, Parish CR (1990). Evidence that platelet and tumour heparanases are similar enzymes. Biochem J.

[2] Vlodavsky I, Friedmann Y (2001). Molecular properties and involvement of heparanase in cancer metastasis and angiogenesis. J Clin Invest.

[3] Nakajima M, Irimura T, Nicolson GL (1988). Heparanases and tumor metastasis. J Cell Biochem.

[4] Bashkin P, Razin E, Eldor A, Vlodavsky I (1990). Degranulating mast cells secrete an endoglycosidase that degrades heparan sulfate in subendothelial extracellular matrix. Blood.

[5] Valentina M, Gianluigi Z, Maria FS, Giovanni G, Antonio L, Maurizio O (2014). Heparanase is a key player in renal fibrosis by regulating TGF-β expression and activity. Biochim Biophys Acta.

[6] Valentina M, Giovanni G, Elena T, Anna MB, Alessandra G, Angela D’A (2012). Heparanase and syndecan-1 interplay orchestrates fibroblast growth factor-2-induced epithelial-mesenchymal transition in renal tubular cells. J Biol Chem.

[7] Shahid S, Caroline F, Simon D, Shahsoltan M, Genevieve C, Cynthia P (2018). The close relationship between heparanase and epithelial mesenchymal transition in gastric signet-ring cell adenocarcinoma. Oncotarget.

[8] Vlodavsky I, Ilan N, Naggi A, Casu B (2007). Heparanase: structure, biological functions, and inhibition by heparin-derived mimetics of heparan sulfate. Curr Pharm Des.

[9] Parish CR (2006). The role of heparan sulphate in inflammation. Nat Rev Immunol.

[10] Vlodavsky I, Singh P, Boyango I, Gutter-Kapon L, Elkin M, Sanderson RD (2016). Heparanase: from basic research to therapeutic applications in cancer and inflammation. Drug Resist Updat.

[11] Zhang GL, Gutter-Kapon L, Ilan N, Batool T, Singh K, Digre A (2020). Significance of host heparanase in promoting tumor growth and metastasis. Matrix Biol.

[12] Bhattacharya U, Gutter-Kapon L, Kan T, Boyango I, Barash U, Yang SM (2020). Heparanase and chemotherapy synergize to drive macrophage activation and enhance tumor growth. Cancer Res.

[13] Zetser A, Bashenko Y, Edovitsky E, Levy-Adam F, Vlodavsky I, Ilan N (2006). Heparanase induces vascular endothelial growth factor expression: correlation with p38 phosphorylation levels and Src ac tivation. Cancer Res.

[14] Koganti R, Suryawanshi R, Shukla D (2020). Heparanase, cell signaling, and viral infections. Cell Mol Life Sci.

[15] He YQ, Sutcliffe EL, Bunting KL, Li J, Goodall KJ, Poon IK (2012). The endoglycosidase heparanase enters the nucleus of T lymphocytes and modulates H3 methylation at actively transcribed genes via the interplay with key chromatin modifying enzymes. Transcription..

[16] Nadir Y, Brenner B, Zetser A, Ilan N, Shafat I, Zcharia E (2006). Heparanase induces tissue factor expression in vascular endothelial and cancer cells. J Thromb Haemost.

[17] Gingis-Velitski S, Zetser A, Flugelman MY, Vlodavsky I, Ilan N (2004). Heparanase induces endothelial cell migration via protein kinase B/Akt activation. J Biol Chem.

[18] Riaz A, Ilan N, Vlodavsky I, Li JP, Johansson S (2013). Characterization of heparanase-induced phosphatidylinositol 3-kinase-AKT activation and its integrin dependence. J Biol Chem.

[19] Cohen-Kaplan V, Doweck I, Naroditsky I, Vlodavsky I, Ilan N (2008). Heparanase augments epidermal growth factor receptor phosphorylation: correlation with head and neck tumor progression. Cancer Res.

[20] Cohen-Kaplan V, Jrbashyan J, Yanir Y, Naroditsky I, Ben-Izhak O, Ilan N (2021). Heparanase induces signal transducer and activator of transcription (STAT) protein phosphorylation: preclinical and clinical significance in head and neck cancer. J Biol Chem.

[21] Levine B, Kroemer G (2008). Autophagy in the pathogenesis of disease. Cell.

[22] Tian X, Teng J, Chen J (2021). New insights regarding SNARE proteins in autophagosome-lysosome fusion. Autophagy.

[23] Xia Y, Liu N, Xie X, Bi G, Ba H, Li L (2019). The macrophage-specific V-ATPase subunit ATP6V0D2 restricts inflammasome activation and bacterial infection by facilitating autophagosome-lysosome fusion. Autophagy.

[24] Kimmelman AC, White E (2017). Autophagy and tumor metabolism. Cell Metab.

[25] Levine B, Kroemer G (2019). Biological functions of autophagy genes: a disease perspective. Cell..

[26] White E, Mehnert JM, Chan CS (2015). Autophagy, metabolism, and cancer. Clin Cancer Res.

[27] Kumar S, Jain A, Choi SW, da Silva GPD, Allers L, Mudd MH (2020). Mammalian Atg8 proteins and the autophagy factor IRGM control mTOR and TFEB at a regulatory node critical for responses to pathogens. Nat Cell Biol.

[28] Guo H, Pu M, Tai Y, Chen Y, Lu H, Qiao J (2021). Nuclear miR-30b-5p suppresses TFEB-mediated lysosomal biogenesis and autophagy. Cell Death Differ.

[29] Li Y, Hodge J, Liu Q, Wang J, Wang Y, Evans TD (2020). TFEB is a master regulator of tumor-associated macrophages in breast cancer. J Immunother Cancer.

[30] Xiang H, Zhang J, Lin C, Zhang L, Liu B, Ouyang L (2020). Targeting autophagy-related protein kinases for potential therapeutic purpose. Acta Pharm Sin B.

[31] He R, Wang M, Zhao C, Shen M, Yu Y, He L (2019). TFEB-driven autophagy potentiates TGF-β induced migration in pancreatic cancer cells. J Exp Clin Cancer Res.

[32] Shteingauz A, Boyango I, Naroditsky I, Hammond E, Gruber M, Doweck I (2015). Heparanase enhances tumor growth and chemoresistance by promoting autophagy. Cancer Res.

[33] Dredge K, Hammond E, Handley P, Gonda TJ, Smith MT, Vincent C (2011). PG545, a dual heparanase and angiogenesis inhibitor, induces potent anti-tumour and anti-metastatic efficacy in preclinical models. Br J Cancer.

[34] Yang M, Pi H, Li M, Xu S, Zhang L, Xie J (2016). From the cover: autophagy induction contributes to cadmium toxicity in mesenchymal stem cells via AMPK/FOXO3a/BECN1 signaling. Toxicol Sci.

[35] Meacham CE, Morrison SJ (2013). Tumour heterogeneity and cancer cell plasticity. Nature.

[36] Barash U, Cohen-Kaplan V, Arvatz G, Gingis-Velitski S, Levy-Adam F, Nativ O (2010). A novel human heparanase splice variant, T5, endowed with protumorigenic characteristics. FASEB J.

[37] Zetser A, Bashenko Y, Miao HQ, Vlodavsky I, Ilan N (2003). Heparanase affects adhesive and tumorigenic potential of human glioma cells. Cancer Res.

[38] Poillet-Perez L, White E (2019). Role of tumor and host autophagy in cancer metabolism. Genes Dev.

[39] Schaaf MB, Keulers TG, Vooijs MA, Rouschop KM (2016). LC3/GABARAP family proteins: autophagy-(un)related functions. FASEB J.

[40] Sarkar C, Zhao Z, Aungst S, Sabirzhanov B, Faden AI, Lipinski MM (2014). Impaired autophagy flux is associated with neuronal cell death after traumatic brain injury. Autophagy..

[41] Kim KH, Lee MS (2014). Autophagy-a key player in cellular and body metabolism. Nat Rev Endocrinol.

[42] Ceccariglia S, Cargnoni A, Silini AR, Parolini O (2020). Autophagy: a potential key contributor to the therapeutic action of mesenchymal stem cells. Autophagy.

[43] Pi H, Li M, Zou L, Yang M, Deng P, Fan T (2019). AKT inhibition-mediated dephosphorylation of TFE3 promotes overactive autophagy independent of MTORC1 in cadmium-exposed bone mesenchymal stem cells. Autophagy..

[44] Schrezenmeier E, Dörner T (2020). Mechanisms of action of hydroxychloroquine and chloroquine: implications for rheumatology. Nat Rev Rheumatol.

[45] Levy JMM, Towers CG, Thorburn A (2017). Targeting autophagy in cancer. Nat Rev Cancer.

[46] Kimura S, Noda T, Yoshimori T (2007). Dissection of the autophagosome maturation process by a novel reporter protein, tandem fluorescent-tagged LC3. Autophagy.

[47] Wang H, Wang N, Xu D, Ma Q, Chen Y, Xu S (2020). Oxidation of multiple MiT/TFE transcription factors links oxidative stress to transcriptional control of autophagy and lysosome biogenesis. Autophagy.

[48] Perera RM, Stoykova S, Nicolay BN, Ross KN, Fitamant J, Boukhali M (2015). Transcriptional control of autophagy-lysosome function drives pancreatic cancer metabolism. Nature.

[49] Purushothaman A, Hurst DR, Pisano C, Mizumoto S, Sugahara K, Sanderson RD (2011). Heparanase-mediated loss of nuclear syndecan-1 enhances histone acetyltransferase (HAT) activity to promote expression of genes that drive an aggressive tumor phenotype. J Biol Chem.

[50] Wang F, Wang Y, Zhang D, Puthanveetil P, Johnson JD, Rodrigues B (2012). Fatty acid-induced nuclear translocation of heparanase uncouples glucose metabolism in endothelial cells. Arterioscler Thromb Vasc Biol.

[51] Raben N, Puertollano R (2016). TFEB and TFE3: linking lysosomes to cellular adaptation to stress. Annu Rev Cell Dev Biol.

[52] Puertollano R, Ferguson SM, Brugarolas J, Ballabio A (2018). The complex relationship between TFEB transcription factor phosphorylation and subcellular localization. EMBO J.

[53] Saftig P, Puertollano R (2021). How lysosomes sense, integrate, and cope with stress. Trends Biochem Sci.

[54] Di Malta C, Siciliano D, Calcagni A, Monfregola J, Punzi S, Pastore N (2017). Transcriptional activation of RagD GTPase controls mTORC1 and promotes cancer growth. Science.

[55] Jiao W, Chen Y, Song H, Li D, Mei H, Yang F (2018). HPSE enhancer RNA promotes cancer progression through driving chromatin looping and regulating hnRNPU/p300/EGR1/HPSE axis. Oncogene.

[56] Boyango I, Barash U, Naroditsky I, Li JP, Hammond E, Ilan N (2014). Heparanase cooperates with Ras to drive breast and skin tumorigenesis. Cancer Res.

[57] Wei RR, Sun DN, Yang H, Yan J, Zhang X, Zheng XL (2018). CTC clusters induced by heparanase enhance breast cancer metastasis. Acta Pharm Sin.

[58] Levy-Adam F, Feld S, Suss-Toby E, Vlodavsky I, Ilan N (2008). Heparanase facilitates cell adhesion and spreading by clustering of cell surface heparan sulfate proteoglycans. PLoS ONE.

[59] Hu X, Zhang L, Jin J, Zhu W, Xu Y, Wu Y (2015). Heparanase released from mesenchymal stem cells activates integrin beta1/HIF-2alpha/Flk-1 signaling and promotes endothelial cell migration and angiogenesis. Stem Cells.

[60] Nadir Y, Brenner B (2016). Heparanase procoagulant activity in cancer progression. Thromb Res.

[61] Crispel Y, Ghanem S, Attias J, Kogan I, Brenner B, Nadir Y (2017). Involvement of the heparanase procoagulant domain in bleeding and wound healing. J Thromb Haemost.

[62] Mizushima N, Levine B (2020). Autophagy in human diseases. N Engl J Med.

[63] Galluzzi L, Green DR (2019). Autophagy-independent functions of the autophagy machinery. Cell.

[64] Mizushima N, Komatsu M (2011). Autophagy: renovation of cells and tissues. Cell.

[65] Behrends C, Sowa ME, Gygi SP, Harper JW (2010). Network organization of the human autophagy system. Nature.

[66] Galluzzi L, Bravo-San Pedro JM, Levine B, Green DR, Kroemer G (2017). Pharmacological modulation of autophagy: therapeutic potential and persisting obstacles. Nat Rev Drug Discov.

[67] Kraya AA, Piao S, Xu X, Zhang G, Herlyn M, Gimotty P (2015). Identification of secreted proteins that reflect autophagy dynamics within tumor cells. Autophagy.

[68] Tsuboyama K, Koyama-Honda I, Sakamaki Y, Koike M, Morishita H, Mizushima N (2016). The ATG conjugation systems are important for degradation of the inner autophagosomal membrane. Science.

[69] Wang Z, Miao G, Xue X, Guo X, Yuan C, Wang Z (2016). The Vici syndrome protein EPG5 is a Rab7 effector that determines the fusion specificity of autophagosomes with late endosomes/lysosomes. Mol Cell.

[70] Takamura A, Komatsu M, Hara T, Sakamoto A, Kishi C, Waguri S (2011). Autophagy-deficient mice develop multiple liver tumors. Genes Dev.

[71] Rosenfeldt MT, O’Prey J, Morton JP, Nixon C, MacKay G, Mrowinska A (2013). p53 status determines the role of autophagy in pancreatic tumour development. Nature.

[72] Yang A, Rajeshkumar NV, Wang X, Yabuuchi S, Alexander BM, Chu GC (2014). Autophagy is critical for pancreatic tumor growth and progression in tumors with p53 alterations. Cancer Discov.

[73] Sardiello M, Palmieri M, di Ronza A, Medina DL, Valenza M, Gennarino VA (2009). A gene network regulating lysosomal biogenesis and function. Science.

[74] Settembre C, Di Malta C, Polito VA, Garcia Arencibia M, Vetrini F, Erdin S (2011). TFEB links autophagy to lysosomal biogenesis. Science.

[75] Yang C, Zhu Z, Tong BC, Iyaswamy A, Xie WJ, Zhu Y (2020). A stress response p38 MAP kinase inhibitor SB202190 promoted TFEB/TFE3-dependent autophagy and lysosomal biogenesis independent of p38. Redox Biol.

[76] Nnah IC, Wang B, Saqcena C, Weber GF, Bonder EM, Bagley D (2019). TFEB-driven endocytosis coordinates MTORC1 signaling and autophagy. Autophagy.

[77] Cinque L, De Leonibus C, Iavazzo M, Krahmer N, Intartaglia D, Salierno FG (2020). MiT/TFE factors control ER-phagy via transcriptional regulation of FAM134B. EMBO J.

[78] López-Hernández T, Puchkov D, Krause E, Maritzen T, Haucke V (2020). Endocytic regulation of cellular ion homeostasis controls lysosome biogenesis. Nat Cell Biol.

[79] Peña-Llopis S, Vega-Rubin-de-Celis S, Schwartz JC, Wolff NC, Tran TA, Zou L (2011). Regulation of TFEB and V-ATPases by mTORC1. EMBO J.

[80] Medina DL, Di Paola S, Peluso I, Armani A, De Stefani D, Venditti R (2015). Lysosomal calcium signalling regulates autophagy through calcineurin and TFEB. Nat Cell Biol.

[81] Settembre C, Zoncu R, Medina DL, Vetrini F, Erdin S, Erdin S (2021). A lysosome-to-nucleus signalling mechanism senses and regulates the lysosome via mTOR and TFEB. EMBO J.

[82] Martina JA, Chen Y, Gucek M, Puertollano R (2012). MTORC1 functions as a transcriptional regulator of autophagy by preventing nuclear transport of TFEB. Autophagy.

[83] Vega-Rubin-de-Celis S, Peña-Llopis S, Konda M, Brugarolas J (2017). Multistep regulation of TFEB by MTORC1. Autophagy.

[84] Martina JA, Diab HI, Lishu L, Jeong AL, Patange S, Raben N (2014). The nutrient-responsive transcription factor TFE3 promotes autophagy, lysosomal biogenesis, and clearance of cellular debris. Sci Signal.

[85] Haq R, Fisher DE (2011). Biology and clinical relevance of the micropthalmia family of transcription factors in human cancer. J Clin Oncol.

[86] Kauffman EC, Ricketts CJ, Rais-Bahrami S, Yang Y, Merino MJ, Bottaro DP (2014). Molecular genetics and cellular features of TFE3 and TFEB fusion kidney cancers. Nat Rev Urol.

[87] Alderton GK (2015). Autophagy: Surviving stress in pancreatic cancer. Nat Rev Cancer.

