# Correction: Non-enzymatic heparanase enhances gastric tumor proliferation via TFEB-dependent autophagy

**DOI:** 10.1038/s41389-023-00487-x

**Published:** 2023-08-18

**Authors:** Min Yang, Bo Tang, Sumin Wang, Li Tang, Dalin Wen, Israel Vlodavsky, Shi-Ming Yang

**Affiliations:** 1 Department of Gastroenterology, Xinqiao Hospital, Army Medical University, Chongqing, 400037 China; 2 Wound Trauma Medical Center, State Key Laboratory of Trauma, Burns and Combined Injury, Daping Hospital, Army Medical University, Chongqing, 400042 China; 3 Cancer and Vascular Biology Research Center, The Bruce Rappaport Faculty of Medicine, Technion, 31096 Haifa, Israel

**Keywords:** Gastric cancer, Autophagy

Correction to: *Oncogenesis* 10.1038/s41389-022-00424-4, published online 15 August 2022

We would like to change the representative image of E225/343A group in Fig. 1g (Attached with corrected image).
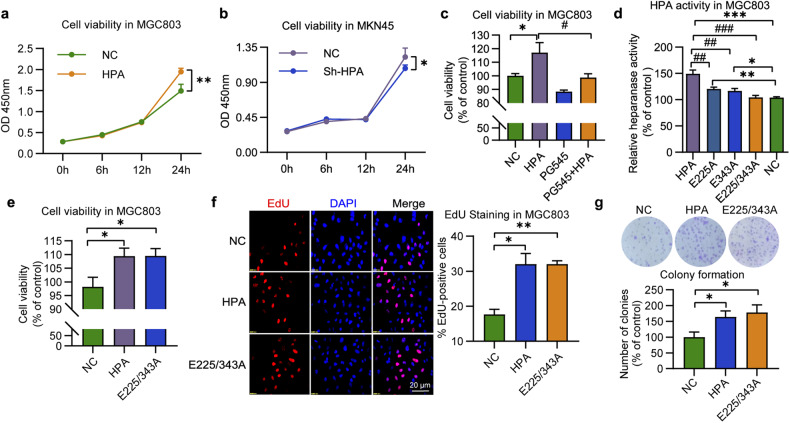


The original article has been corrected.

