# Supplementary material for: Non-enzymatic heparanase enhances gastric tumor proliferation via TFEB-dependent autophagy
Source: Oncogenesis. 2022 Aug 15;11(1):49. doi: 10.1038/s41389-022-00424-4 (PMC9378687; doi:10.1038/s41389-022-00424-4)
Supplement: Supplementary file 1 — Supplemental figures [file 41389_2022_424_MOESM1_ESM.docx]

**Supplemental Figures**


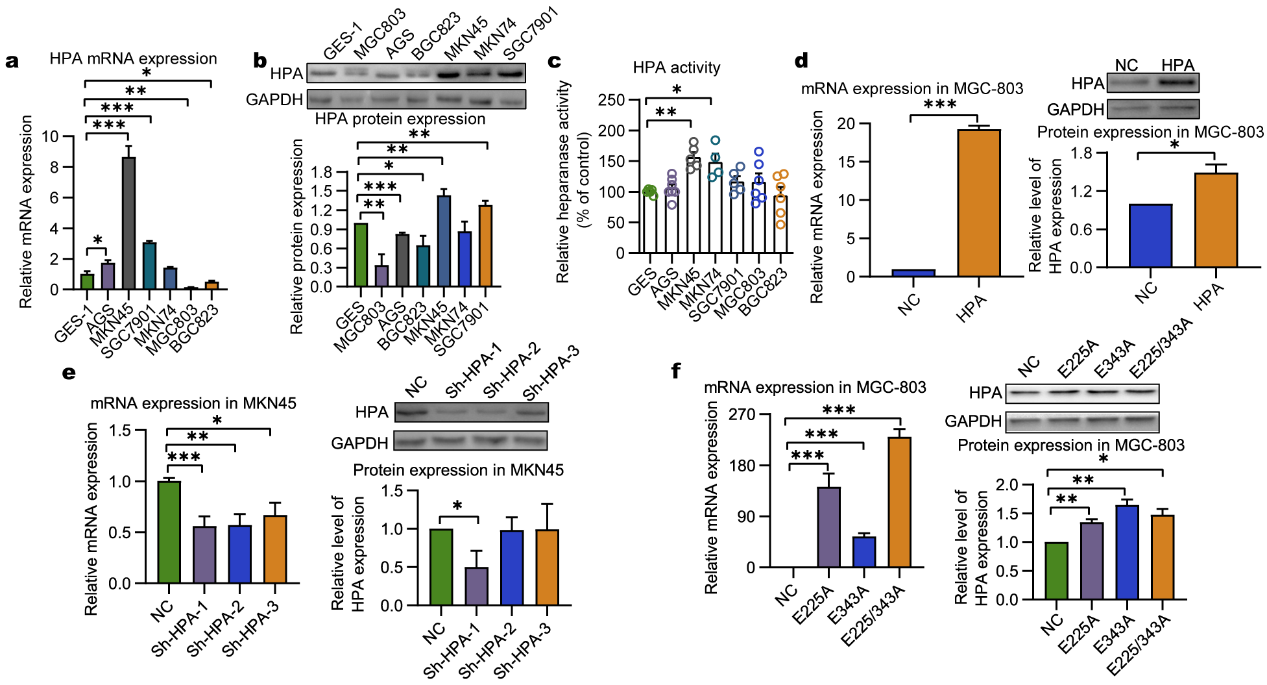


**Supplementary Figure 1: effects of HPA overexpression and knockdown in gastric cancer cell lines.** (**a**) qPCR analysis of HPA mRNA and (**b**) western blot analysis of HPA protein and (**c**) HTRF assay analysis of HPA enzymatic activity in a normal human gastric epithelial cell line (GES-1) and six GC cell lines. mRNA levels and representative images of HPA proteins in MGC803 cells after overexpressed (**d**) and mutant HPA plasmid (**e**) transfection and in MKN45 cells after HPA knockdown (**f**). Data are presented as the mean ± SEM, * P<0.05, ** P<0.01, *** P<0.001 vs the GES-1 or NC group.


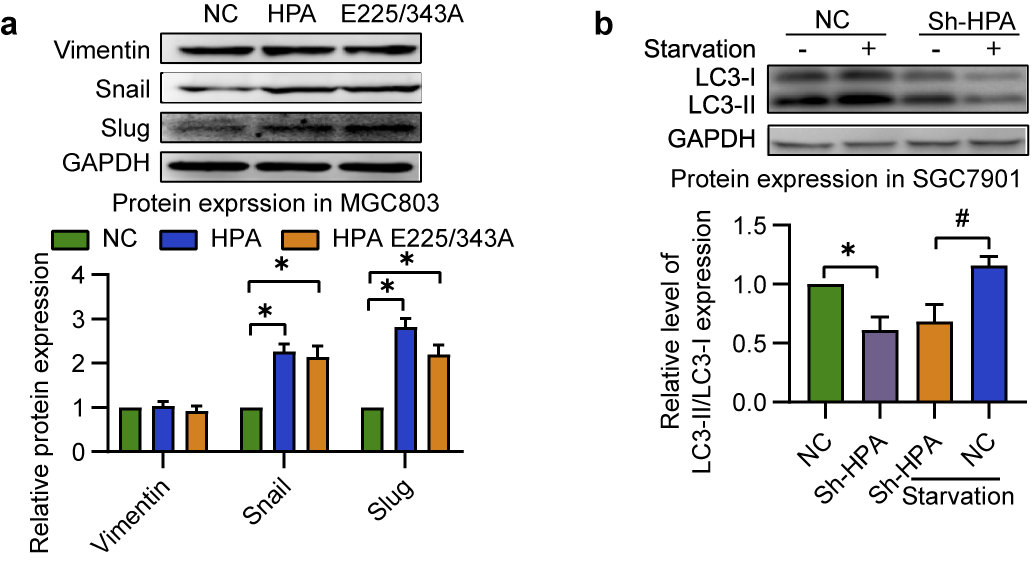


**Supplementary Figure 2：EMT makers expression in MGC803 cells and LC3-II protein in SCG7901 cells.** (**a**) Representative image and quantification analysis of EMT-related protein expression in MGC803 cells after wild type or mutant HPA transfection. (**b**) Representative image and quantification analysis of and LC3-II in SCG7901 cells after HPA knock-down with or without starvation. GAPDH was used as a loading control. Data are presented as the mean ± SEM, * P<0.05 versus the NC group, # P<0.05 versus the NC + starvation group.


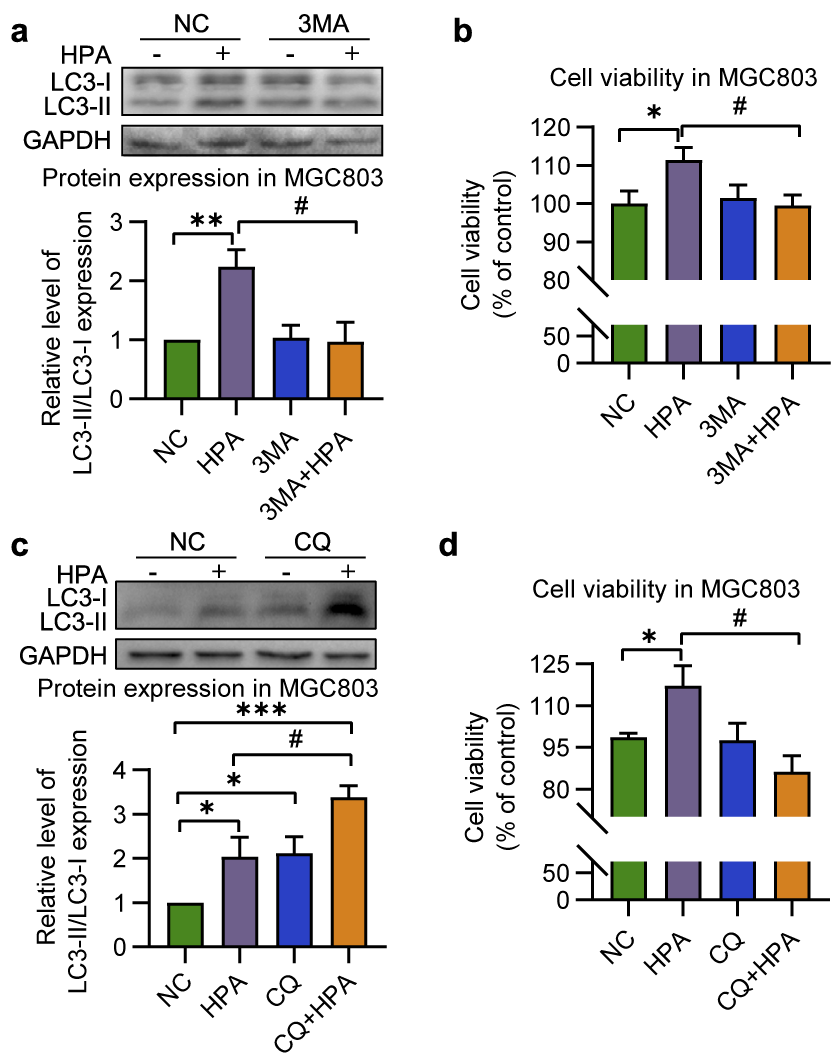


**Supplementary Figure 3：HPA-induced LC3 protein abundant and cell viability was reversed by autophagy inhibitor.** Western blot analysis of LC3-II (**a**) and cell viability (**b**) in MGC803 cells after wild type HPA transfection in the absence or presence of 3MA. representative image and quantification of LC3-II protein level (**c**) and cell proliferation (**d**) in MGC803 cells treated with HPA overexpression plasmids with or without CQ. GAPDH was used as a loading control. Data are presented as the mean ± SEM, * P<0.05, ** P<0.01, *** P<0.001 versus the NC group, # P<0.05 versus the HPA group.


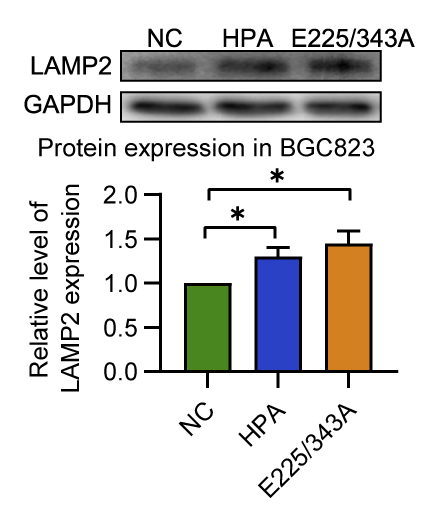


**Supplementary Figure 4：both wild type and non-enzymatic HPA aggravated LAMP2 protein in BGC823 cells.** LAMP2 protein expression and quantification in BGC823 cells after transfection with overexpressed or mutant HPA, in the condition of starvation. GAPDH was used as a loading control. Data are presented as the mean ± SEM, * P<0.05 versus the NC group.


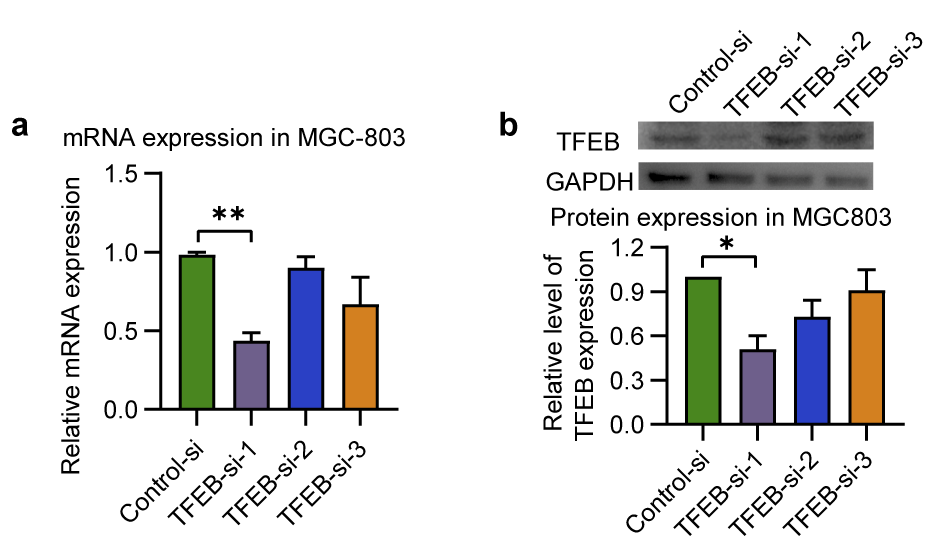


**Supplementary Figure 5：Gene silence effect of TFEB in MCG803 cells after TFEB gene knock down.** (**a**) relative gene expression of TFEB in MGC803 cells after TFEB siRNA transfection. (**b**) Representative image and quantification of TFEB protein in MGC 803 cells after TFEB gene knock down. Data are presented as the mean ± SEM, * P<0.05, ** P<0.01 versus the Control-si group.
