# Supplementary material for: Non-enzymatic heparanase enhances gastric tumor proliferation via TFEB-dependent autophagy
Source: Oncogenesis. 2022 Aug 15;11(1):49. doi: 10.1038/s41389-022-00424-4 (PMC9378687; doi:10.1038/s41389-022-00424-4)
Supplement: Supplementary file 2 — Table S1 [file 41389_2022_424_MOESM2_ESM.docx]

**Table S1:** the origins of the cells used

| Name | Differentiation degree | Source |
| --- | --- | --- |
| AGS | Moderately differentiated | Chinese Academy of Sciences |
| BGC823 | poor differentiation | Chinese Academy of Sciences |
| SGC7901 | poor differentiation | Chinese Academy of Sciences |
| MKN45 | poor differentiation | Chinese Academy of Sciences |
| MGC803 | poor differentiation | Chinese Academy of Sciences |
| MKN74 | Well differentiated | Chinese Academy of Sciences |
| GES-1 | Normal epithelial cell | Chinese Academy of Sciences |
