# Supplementary material for: Non-enzymatic heparanase enhances gastric tumor proliferation via TFEB-dependent autophagy
Source: Oncogenesis. 2022 Aug 15;11(1):49. doi: 10.1038/s41389-022-00424-4 (PMC9378687; doi:10.1038/s41389-022-00424-4)
Supplement: Supplementary file 3 — Table S2 [file 41389_2022_424_MOESM3_ESM.docx]

**Table S2:** Sequences of human primers used in quantitative RT-PCR

| **Target gene** | **Primer** | **Nucleotide sequence** |
| --- | --- | --- |
| HPA1 | Forward | 5’-CGGCTAAGATGCTGAAGAGC-3’ |
|  | Reverse | 5’-TGATGCCATGTAACTGAATCAA-3’ |
| TFEB | Forward | 5’-GGTGCAGTCCTACCTGGAGA-3’ |
|  | Reverse | 5’-GTGGGCAGCAAACTTGTTCC-3’ |
| TFE3 | Forward | 5’-GAACAAGGGCACCATCCTGA-3’ |
|  | Reverse | 5’-TGGGCCTGCAGTTCTAGTTC-3’ |
| TFEC | Forward | 5’-TTAGAGCAGGCTAACAGGCG-3’ |
|  | Reverse | 5’-ATGGCTCTGCTGTTTGGTGA-3’ |
| MITF | Forward | 5’-GGGCTTGATGGATCCTGCTT-3’ |
|  | Reverse | 5’-ATGTTGGGAAGGTTGGCTGG-3’ |
| FIP200 | Forward | 5’-ATCGAAGAGTGTGTACCTACAGT-3’ |
|  | Reverse | 5’-GCAGGTGGACGATCACATAAGAT-3’ |
| ATG4A | Forward | 5’-TTCTTGGACCCTCATACAACCC-3’ |
|  | Reverse | 5’-TTAGGATGTTCATTCGCTGTGG-3’ |
| ATG13 | Forward | 5’-AGACAGTTCGTGTTGGGACAG-3’ |
|  | Reverse | 5’-CTCAAATTGCCTGGTAGACATGA-3’ |
| UVRAG | Forward | 5’-ATGCCAGACCGTCTTGATACA-3’ |
|  | Reverse | 5’-TGACCCAAGTATTTCAGCCCA-3’ |
| VPS34 | Forward | 5’-CCTGGAAGACCCAATGTTGAAG-3’ |
|  | Reverse | 5’-CGGGACCATACACATCCCAT-3’ |
| ATG14 | Forward | 5’-GCGCCAAATGCGTTCAGAG-3’ |
|  | Reverse | 5’-AGTCGGCTTAACCTTTCCTTCT-3’ |
| ATG4B | Forward | 5’-GGTGTGGACAGATGATCTTTGC-3’ |
|  | Reverse | 5’-CCAACTCCCATTTGCGCTATC-3’ |
| ATG3 | Forward | 5’-ACATGGCAATGGGCTACAGG-3’ |
|  | Reverse | 5’-CTGTTTGCACCGCTTATAGCA-3’ |
| ATG10 | Forward | 5’-AGACCATCAAAGGACTGTTCTGA-3’ |
|  | Reverse | 5’-GGGTAGATGCTCCTAGATGTGAC-3’ |
| ATG12 | Forward | 5’-TAGAGCGAACACGAACCATCC-3’ |
|  | Reverse | 5’-CACTGCCAAAACACTCATAGAGA-3’ |
| ATG16L1 | Forward | 5’-AACGCTGTGCAGTTCAGTCC-3’ |
|  | Reverse | 5’-AGCTGCTAAGAGGTAAGATCCA-3’ |
| ULK1 | Forward | 5’-GGCAAGTTCGAGTTCTCCCG-3’ |
|  | Reverse | 5’-CGACCTCCAAATCGTGCTTCT-3’ |
| P62 | Forward | 5’-GACTACGACTTGTGTAGCGTC-3’ |
|  | Reverse | 5’-AGTGTCCGTGTTTCACCTTCC-3’ |
| BECN1 | Forward | 5’-ACCTCAGCCGAAGACTGAAG-3’ |
|  | Reverse | 5’-AACAGCGTTTGTAGTTCTGACA-3’ |
| ATG5 | Forward | 5’-AGAAGCTGTTTCGTCCTGTGG-3’ |
|  | Reverse | 5’-AGGTGTTTCCAACATTGGCTC-3’ |
| ATG7 | Forward | 5’-ATGATCCCTGTAACTTAGCCCA-3’ |
|  | Reverse | 5’-CACGGAAGCAAACAACTTCAAC-3’ |
| LC3 | Forward | 5’-AACATGAGCGAGTTGGTCAAG-3’ |
|  | Reverse | 5’-GCTCGTAGATGTCCGCGAT-3’ |
| AGA | Forward | 5’-CGGAAGTCGAACTTGCCTGT-3’ |
|  | Reverse | 5’-TCGGTTGCATTCTTAAAGGGC-3’ |
| ARSA | Forward | 5’-CACACCCACTACCCTCAGTTC-3’ |
|  | Reverse | 5’-CAGGTCCCCTATGGCTGTC-3’ |
| ARSB | Forward | 5’-TCTTGCTGGCAGACGACCTA-3’ |
|  | Reverse | 5’-GGCTGCGTGTAGTAGTTGTCC-3’ |
| ATP6V0E1 | Forward | 5’-GTCCTAACCGGGGAGTTATCA-3’ |
|  | Reverse | 5’-AAAGAGAGGGTTGAGTTGGGC-3’ |
| ATP6V1H | Forward | 5’-CAGAAGTTCGTGCAAACAAAGTC-3’ |
|  | Reverse | 5’-TCAGGGCTTCGTTTCATTTCAA-3’ |
| CLCN7 | Forward | 5’-CCCACACAACGAGAAGCTCC-3’ |
|  | Reverse | 5’-ACTTGTCGATATTGCCCTTGATG-3’ |
| CTSA | Forward | 5’-GTCGCCCAGAGCAATTTTGAG-3’ |
|  | Reverse | 5’-TCTCCCCGGTCAGGAAAAGTT-3’ |
| CTSB | Forward | 5’-GAGCTGGTCAACTATGTCAACA-3’ |
|  | Reverse | 5’-GCTCATGTCCACGTTGTAGAAGT-3’ |
| CTSD | Forward | 5’-TGCTCAAGAACTACATGGACGC-3’ |
|  | Reverse | 5’-CGAAGACGACTGTGAAGCACT-3’ |
| CTSF | Forward | 5’-AGCCCAAGTCAGCCTTCAC-3’ |
|  | Reverse | 5’-CGCACCATGTTATTGACAAAGAC-3’ |
| GALNS | Forward | 5’-GTGACCTCGGGGTGTATGGA-3’ |
|  | Reverse | 5’-AAGCCATTGCGGATGGGTAG-3’ |
| GBA | Forward | 5’-CATCCGCACCTACACCTATGC-3’ |
|  | Reverse | 5’-TGAGCTTGGTATCTTCCTCTGG-3’ |
| GLA | Forward | 5’-CTGAGGAACCCAGAACTACATCT-3’ |
|  | Reverse | 5’-GGTAGGCGTCCTTGCCAAT-3’ |
| GNS | Forward | 5’-GCATGACACCGCTAAAGAAAAC-3’ |
|  | Reverse | 5’-CACAACGTGATGATTATGTGGGT-3’ |
| HEXA | Forward | 5’-ACGTCCTTTACCCGAACAACT-3’ |
|  | Reverse | 5’-CGAAAAGCAGGTCACGATAGC-3’ |
| LAMP1 | Forward | 5’-TCTCAGTGAACTACGACACCA-3’ |
|  | Reverse | 5’-AGTGTATGTCCTCTTCCAAAAGC-3’ |
| LAMP2 | Forward | 5’-GAAAATGCCACTTGCCTTTATGC-3’ |
|  | Reverse | 5’-AGGAAAAGCCAGGTCCGAAC-3’ |
| MCOLN1 | Forward | 5’-TTCGCCGTCGTCTCAAATACT-3’ |
|  | Reverse | 5’-CTCTTCCCGGAATGTCACAGC-3’ |
| NAGLU | Forward | 5’-ACCGCTATTACCAGAATGTGTG-3’ |
|  | Reverse | 5’-CCATCCAGTCTATCTCTCGCTC-3’ |
| NEU1 | Forward | 5’-GGAGGCTGTAGGGTTTGGG-3’ |
|  | Reverse | 5’-CACCAGACCGAAGTCGTTCT-3’ |
| PSAP | Forward | 5’-CCCGGTCCTTGGACTGAAAG-3’ |
|  | Reverse | 5’-TATGTCGCAGGGAAGGGATTT-3’ |
| SCPEP1 | Forward | 5’-CTGAACGCAGGAGCTGTCATT-3’ |
|  | Reverse | 5’-CCTTGCGGACCGTCACATAAT-3’ |
| SGSH | Forward | 5’-ACGGAGGCTTTGAGAGTGG-3’ |
|  | Reverse | 5’-GCATTGCGAAAGAGGAGGCT-3’ |
| TPP1 | Forward | 5’-CCTCCACACGGTGCAAAAATG-3’ |
|  | Reverse | 5’-CTCTGCTTGTCGGATGCTCAG-3’ |
| TMEM55B | Forward | 5’-GACAGTGGGAGTGCCCCTAT-3’ |
|  | Reverse | 5’-GGGGTGCATTCTTGATTGGG-3’ |
| ACTB | Forward | 5’-GCACTCTTCCAGCCTTCCTT-3’ |
|  | Reverse | 5’-CGTACAGGTCTTTGCGGATG -3’ |
