# Supplementary material for: Non-enzymatic heparanase enhances gastric tumor proliferation via TFEB-dependent autophagy
Source: Oncogenesis. 2022 Aug 15;11(1):49. doi: 10.1038/s41389-022-00424-4 (PMC9378687; doi:10.1038/s41389-022-00424-4)
Supplement: Supplementary file 4 — Table S3 [file 41389_2022_424_MOESM4_ESM.docx]

**Table S3:** Antibodies used for the western blot experiments

| Antigen | Dilution | Catalogue number | Supplier |
| --- | --- | --- | --- |
| TFEB | 1:1000 | 37785 | Cell Signaling Technology |
| p-TFEB (Ser211) | 1:1000 | 37681 | Cell Signaling Technology |
| TFE3 | 1:1000 | 14779 | Cell Signaling Technology |
| BECN1 | 1:1000 | 3495 | Cell Signaling Technology |
| LC3 | 1:1000 | Ab51520 | Abcam |
| LAMP2 | 1:1000 | Ab25631 | Abcam |
| HPA1 | 1:1000 | Sc-515935 | Santa Cruz |
| 14-3-3 | 1:1000 | 9636S | Cell Signaling Technology |
| EMT antibody Sample Kit | 1:1000 | 9782 | Cell Signaling Technology |
| Lamin B1 | 1:1000 | 12987-1-AP | Proteintech |
| GAPDH | 1:1000 | Sc-47724 | Santa Cruz |
| Anti-mouse (secondary antibody) | 1:2000 | A0208 | Beyotime Company |
| Anti-rabbit (secondary antibody) | 1:2000 | A0216 | Beyotime Company |
