# Supplementary material for: Non-enzymatic heparanase enhances gastric tumor proliferation via TFEB-dependent autophagy
Source: Oncogenesis. 2022 Aug 15;11(1):49. doi: 10.1038/s41389-022-00424-4 (PMC9378687; doi:10.1038/s41389-022-00424-4)
Supplement: Supplementary file 5 — Supplemental data 1 [file 41389_2022_424_MOESM5_ESM.pdf]

Expression of HPSE in STAD based on Sample types

TCGA samples	Series 1				
	**low**	**q1**	**median**	**q3**	**high**
**Normal (n=34)**	0.08	0.50	2.02	3.14	5.52
**Primary tumor (n=415)**	0.17	3.83	6.47	10.23	22.61
