# Supplementary material for: Non-enzymatic heparanase enhances gastric tumor proliferation via TFEB-dependent autophagy
Source: Oncogenesis. 2022 Aug 15;11(1):49. doi: 10.1038/s41389-022-00424-4 (PMC9378687; doi:10.1038/s41389-022-00424-4)
Supplement: Supplementary file 7 — Supplemental data 3 [file 41389_2022_424_MOESM7_ESM.pdf]

Expression of HPSE in STAD based on nodal metastasis status

TCGA samples	Series 1				
	low	q1	median	q3	high
Normal (n=34)	0.08	0.50	2.02	3.14	5.52
N0 (n=123)	0.17	3.89	5.66	9.67	19.37
N1 (n=112)	1.03	4.50	6.95	10.73	21.78
N2 (n=79)	0.28	3.67	6.17	9.59	18.38
N3 (n=82)	0.47	3.59	7.26	11.83	21.82
