# Supplementary material for: Non-enzymatic heparanase enhances gastric tumor proliferation via TFEB-dependent autophagy
Source: Oncogenesis. 2022 Aug 15;11(1):49. doi: 10.1038/s41389-022-00424-4 (PMC9378687; doi:10.1038/s41389-022-00424-4)
Supplement: Supplementary file 8 — author list change [file 41389_2022_424_MOESM8_ESM.pdf]

SPRINGER NATURE

In accordance with Springer Nature Authorship Policy we agree to change the authors of the manuscript as indicated below.

NAME OF JOURNAL: Oncogenesis

TITLE OF MANUSCRIPT: Non-enzymatic heparanase enhances gastric tumor proliferation via TFEB-dependent autophagy

MANUSCRIPT NUMBER: ONCSIS-22-0044

CORRESPONDING AUTHORS NAME: Shi-Ming Yang

PREVIOUS AUTHOR NAMES:

Min Yang, Bo Tang, Li Tang, Sumin Wang, Dalin Wen, Israel Vlodavsky, Shi-Ming Yang

UPDATED AUTHOR NAMES:

CHANGE TO AUTHOR LIST:

Min Yang, Bo Tang, Sumin Wang, Li Tang, Dalin Wen, Israel Vlodavsky, Shi-Ming Yang

Print Name	Signature	Date
Min Yang	Min Yang	2022.6.1
Bo Tang	Bo Tang	2022.6.1
Sumin Wang	Sumin Wang	2022.6.1
Li Tang	Li Tang	2022.6.1
Dalin Wen	Dalin Wen	2022.6.1
Israel Vlodavsky	Israel Vlodavsky	2022.6.1
Shi-Ming Yang	Shi-Ming Yang	2022.6.1
